# Evidence for Composite Cost Functions in Arm Movement Planning: An Inverse Optimal Control Approach

**DOI:** 10.1371/journal.pcbi.1002183

**Published:** 2011-10-13

**Authors:** Bastien Berret, Enrico Chiovetto, Francesco Nori, Thierry Pozzo

**Affiliations:** 1 Italian Institute of Technology, Department of Robotics, Brain and Cognitive Sciences, Genoa, Italy; 2 University Clinic Tübingen, Section for Computational Sensomotorics, Department of Cognitive Neurology, Hertie Institute of Clinical Brain Research and Center for Integrative Neurosciences, Tübingen, Germany; 3 Institut Universitaire de France, Université de Bourgogne, Campus Universitaire, UFR STAPS, Dijon, France; 4 INSERM, U887, Motricité-Plasticité, Dijon, France; University College London, United Kingdom

## Abstract

An important issue in motor control is understanding the basic principles underlying the accomplishment of natural movements. According to optimal control theory, the problem can be stated in these terms: what cost function do we optimize to coordinate the many more degrees of freedom than necessary to fulfill a specific motor goal? This question has not received a final answer yet, since what is optimized partly depends on the requirements of the task. Many cost functions were proposed in the past, and most of them were found to be in agreement with experimental data. Therefore, the actual principles on which the brain relies to achieve a certain motor behavior are still unclear. Existing results might suggest that movements are not the results of the minimization of single but rather of composite cost functions. In order to better clarify this last point, we consider an innovative experimental paradigm characterized by arm reaching with target redundancy. Within this framework, we make use of an inverse optimal control technique to automatically infer the (combination of) optimality criteria that best fit the experimental data. Results show that the subjects exhibited a consistent behavior during each experimental condition, even though the target point was not prescribed in advance. Inverse and direct optimal control together reveal that the average arm trajectories were best replicated when optimizing the combination of two cost functions, nominally a mix between the absolute work of torques and the integrated squared joint acceleration. Our results thus support the cost combination hypothesis and demonstrate that the recorded movements were closely linked to the combination of two complementary functions related to mechanical energy expenditure and joint-level smoothness.

## Author Summary

To reach an object, the brain has to select among a set of possible arm trajectories that displace the hand from an initial to a final desired position. Because of the intrinsic redundancy characterizing the human arm, the number of admissible joint trajectories toward the goal is generally infinite. However, many studies have demonstrated that the range of actual trajectories can be limited to those that result from the fulfillment of some optimal rules. Various cost functions were shown to be relevant in the literature. A peculiar aspect of most of these costs is that each one of them aims at optimizing one specific feature of the movement. The necessary motor flexibility of everyday life, however, might rely on the combination of such cost functions rather than on a single one. Testing this cost combination hypothesis has never been attempted. To this aim we propose a reaching task involving target redundancy to facilitate the comparisons of different candidate costs and to identify the best-fitting one (possibly composite). Using a numerical inverse optimal control method, we show that most participants produced movements corresponding to a strict combination of two subjective costs linked to the mechanical energy consumption and the joint-level smoothness.

## Introduction

Numerous experimental studies have demonstrated that biological motion exhibits invariant features, i.e. parameters that do not significantly change with movement size, speed, load and direction [1]–[4]. A number of these features was described for point-to-point (e.g. reaching, see [5]) and continuous (e.g. drawing and handwriting, see [6]) movements of the upper limb. Therefore, despite the infinite number of motor strategies compatible with most of these tasks, regularities characterize human voluntary movements, suggesting that the central nervous system (CNS) overcomes the redundancy of movement accomplishment by following some specific rules or principles. Many authors investigated these principles in the framework of deterministic optimal control theory. This theory assumes that biological movements are optimal in the sense that they minimize some performance criteria or cost/loss functions. In this regard, a plethora of optimal control models have been proposed in the literature [7, 8, for reviews] and most of them were found to fit well the experimental data. Therefore, the exact relationship between different mathematical cost functions and the movement variables actually represented in the brain still remains unclear and this seems due to multiple reasons.

The first one is methodological: in many cases, models based on divergent assumptions and minimizing different costs can yield similar arm trajectories [9], [10]. If one considers the range of human motor variability and the consequences of model approximations, several cost functions can perform well enough to be considered valid. For example, the minimum hand jerk [11], the minimum torque change [12] but also the minimum variance models [13] make fully acceptable predictions for point-to-point arm movements performed in the horizontal plane (i.e. quasi-straight hand paths with bell-shaped time-courses). The second reason is conceptual: seeking a single and universal cost function might be useless [10], in particular if the CNS is capable of optimizing a weighted combination of costs depending on the features of the task [14]–[17]. Thus, a part of the present collection of models may represent constituent pieces of one motor plan. It is already well-known for instance that the weight given to objective (e.g. task-related) and subjective (e.g. body-related) cost functions can be modulated by the CNS. Increasing the accuracy requirements of a pointing task while keeping the movement time constant leads to an increase of muscle co-contraction (and thus of metabolic energy expenditure, see [18]). Conversely, experimentally induced fatigue leads to a reweighting of accuracy and energy economy requirements in the sensorimotor control of fast elbow flexions [19]. Hence, cost functions would result in any case from the combination of external task demands with internal constraints. In contrast to this well-identified objective/subjective costs trade-off (see also [20]) it has not been established yet whether or not the CNS actually combines subjective costs (e.g. neural or mechanical energy expenditures, hand/joint/torque jerk, amount of torques/forces etc.).

**Figure 1:**
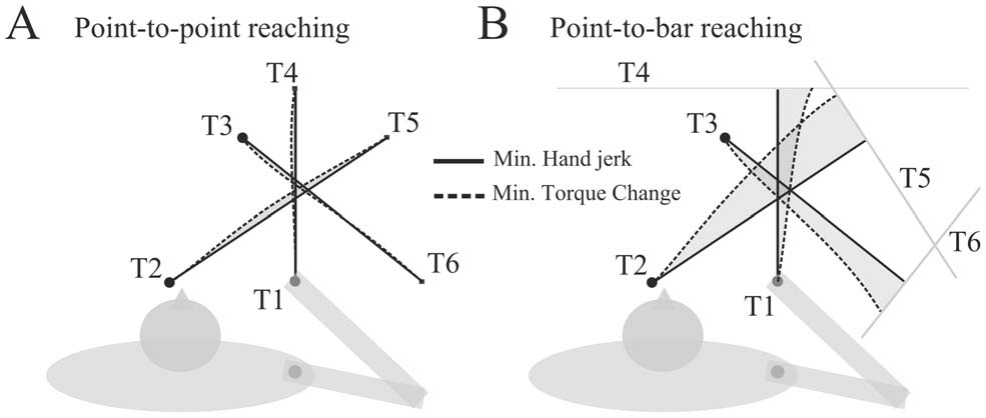
Proof of concept: illustration that the hand jerk and torque change costs are more discernible during reaching to a bar than to a point. A. Simulated hand paths for point-to-point movements in the horizontal plane. Targets (T1 to T6) were located approximately as in [11]. B. Simulated hand paths for the point-to-bar case. The starting points are the same as in panel A, but we replaced the target points by target lines/bars. The shaded areas emphasize the amount of difference between these two cost functions.

In order to test this cost combination hypothesis, our approach was two-fold. First, we wanted to stress the differences between the predictions of different classical models already existing in literature. To this aim, we designed a pointing task with target redundancy. Precisely, we reduced the external constraints of the task by asking subjects to reach to a vertical bar. Thus no accuracy requirement was present in the vertical axis, which had the interesting advantage of discriminating better between different cost functions than during classical point-to-point experiments (see Figure 1 for a proof-of-concept). Second, we developed a framework permitting us to examine simultaneously several existing models/costs, as well as any linear combination of them, by means of an automated inverse optimal control method. Inverse optimal control is a mathematical approach in which inference about the cost function is made automatically from experimental data, which are assumed to be optimal [21]. Using such a method, we were able to link the recorded data to an infinite number of potential (composite) cost functions, in contrast to the a priori choice of one single cost function characterizing most of the previous investigations. In this way we could automatically uncover which single cost or mix of costs fit best with the average behavior of subjects. Direct optimal control was then used to strengthen the results provided by the inverse method and to compare directly the recorded and simulated data.

The experimental results show that participants adopted a consistent behavior although the final point was not imposed by the experimenter. Inverse optimal control reveals that their average behavior mainly relied on a composite cost function, combining the minimization of mechanical energy expenditure (here the absolute work of torques) with the maximization of joint smoothness (here the integrated squared acceleration). Further analyses demonstrate that this mix-of-cost model replicated the most important features of arm movements and performed better than any other single cost function on which our method was based. Results provided therefore support the cost combination hypothesis and, in particular for this task, emphasize two complementary and subjective costs.

## Materials and Methods

### Experimental task

#### Participants

Twenty naive subjects (16 males, and, *meanstd* 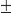 *: age* 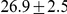, range 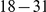; mass 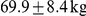; height 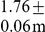) volunteered to participate in the experiment. All of them were healthy, right-handed and with normal or corrected-to-normal vision. Written informed consent was obtained from each participant in the study, which was approved by the local ethical committee ASL-3 (“Azienda Sanitaria Locale”, local health unit), Genoa, and was in agreement with the Helsinki Declaration of 1975, as revised in 1983.

#### Reaching-to-a-bar task

**Figure 2:**
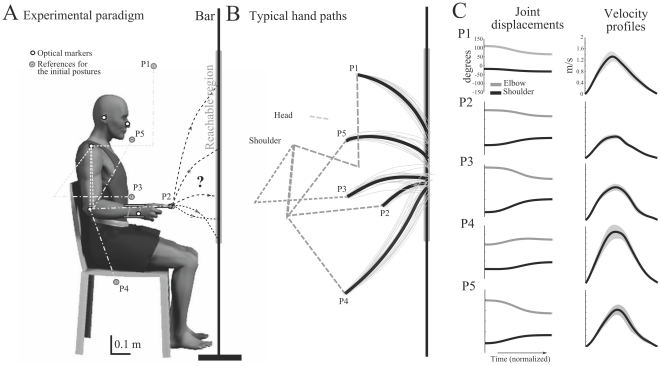
A. Illustration of the experimental paradigm. The reachable region from the sitting position is emphasized on the bar. The 5 initial postures under consideration are also shown (P1 to P5). B. Experimental trajectories for a representative subject. Dotted lines depict the initial arm posture of the subject (upper arm and forearm). The average fingertip path is shown in thick black line for each initial posture, from P1 to P5. The 20 trials are depicted in thin gray lines for every initial postures. C. Experimental angular displacements and finger velocity profiles for the most typical subject. First column: joint displacements at the shoulder and elbow joints; Second column: Finger velocity profiles with shaded areas indicating the standard deviation. Time is normalized, not amplitude.

The motor task that we considered is illustrated in Figure 2A. From a sitting position, participants were asked to perform a series of pointing movements toward a vertical target bar. The bar was a uniform and rigid tube. For the task, shoulder and elbow rotations were allowed, while the wrist joint was frozen by means of two light and small sticks attached to the distal part of the forearm and the proximal part of the hand. The vertical bar was placed in front of the participants, in the para-sagittal plane intersecting the shoulder joint. No target point was emphasized on the bar and its height was 2.50 meters so that subjects could not see its extremities without moving the head or the trunk. The horizontal distance of the shoulder from the bar was set to 85% of the subject's full arm length (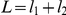, where 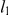 and 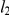 denote the upper arm and forearm lengths respectively, see Figure 2A). Five initial arm postures, denoted by P1 to P5, were defined by means of reference points located in a vertical plane, placed laterally at approximately 10 cm from the subject's right shoulder. Precisely, these five starting postures were defined by imposing the following angular arm configurations ([elbow;shoulder] in degrees): 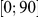, 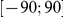, 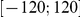, 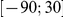 and 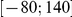, respectively from P1 to P5.

The initial references were positioned using a wooden hollow frame containing 1.5 cm-spaced thin vertical fishing wires to which lead weights (small spheres) indicating the requested fingertip initial position were attached. Differently colored pieces of scotch-tapes were stuck on the leads to easily identify the references. This color-code was then used to verbally specify the initial posture that the subject had to select at the beginning of each movement. By imposing the initial finger position, a unique starting posture of the arm was thus defined in the para-sagittal plane. The positions of the leads were adjusted before the experiment, based on the subject's upper arm and forearm lengths and the vertical distance shoulder-ground.

The experimenter then gave the following instruction to the participants: look at the bar in front of you, close the eyes and quickly show the location of the bar by touching it with the fingertip, performing a one-shot movement. No instruction was given to the subjects with respect to where and how to reach the bar. Because of the features of the task itself, participants had to implicitly control the finger position along the antero-posterior and lateral directions whereas full freedom was left along the vertical one. Note that the challenge for the subjects (i.e. the objective reward of the task) was to be precise enough to actually touch the bar, since no on-line vision was allowed. Since subjects were free to moved in 3-D, touching the bar was not so easy because of the presence lateral and antero-posterior errors and the absence of on-line visual feedback. Nevertheless, it is worth noting that reaching any point on the vertical bar allowed the subject to perform the task successfully. During the protocol, the five initial postures were tested in a random order. For each initial posture, twenty trials were recorded, so that a total of 100 movements per subject were monitored. A few trials were repeated during the experiment (less than 5%), when the subjects clearly missed the bar or did not perform a one-shot movement. Every set of 25 movements, subjects were allowed to rest. Data from a total of 2000 pointing movements were collected for this reaching-to-a-bar task.

### Data collection and processing

#### Materials

Arm and head motion were recorded by means of a motion capture system (Vicon, Oxford, UK). Ten cameras were used to capture the movement of six retro reflective markers (15 mm in diameter), placed at well-defined anatomical locations on the right arm and head (acromial process, humeral lateral condyle, ulnar styloid process, apex of the index finger, external cantus of the eye, and auditory meatus).

#### Motion analysis

All the analyses were performed with custom software written in Matlab (Mathworks, Natick, MA) from the recorded three-dimensional position of the six markers (sampling frequency, 100 Hz). Recorded signals were low-pass filtered using a digital fifth-order Butterworth filter at a cutoff frequency of 10 Hz (Matlab *filtfilt* function).

The temporal finger movement onset was defined as the instant at which the linear tangential velocity of the fingertip exceeded 5% of its peak and the end of movement as the point at which the same velocity dropped below the 5% threshold. All time series were normalized to 200 points by using Matlab routines of interpolation (Matlab *spline* function). Standard kinematic parameters described in previous experimental arm pointing studies were calculated [3], [22]: *movement duration* (MD), *peak velocity* (PV), *mean velocity* (MV), *relative time to peak velocity* (TPV) defined as the ratio between the acceleration duration and MD, *index of velocity shape* (Vpeak/Vmean) defined as the ratio between the peak of velocity and its mean value, and *curvilinear distance* of the finger (CD) defined by the integral over time from 0 to MD of the norm of the fingertip velocity vector. The *constant error* was computed as the orthogonal distance between the terminal finger position and the bar. The *variable error* was defined as the standard deviation computed on the distances between the measured endpoints across trials.

For subsequent analyses and comparisons with models, we projected the 3-D coordinates of the markers onto a vertical plane. It will be shown thereafter that the movements carried out by the participants almost lay on a para-sagittal plane. The motion capture system was calibrated such that the axes 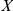 and 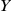 corresponded to the antero-posterior and vertical axes, respectively. Thus, movements were approximately in the 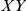 plane, while the 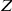 direction (lateral) was not significantly used.

Angular displacements of the arm segments (upper arm and forearm) were then evaluated using the inverse kinematic function, relating the 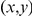 position of the finger in plane 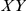 to the arm configuration 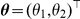 (subscript 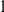 denoting the shoulder joint):
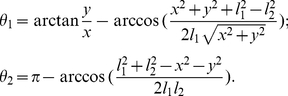
(1)


**Figure 3:**
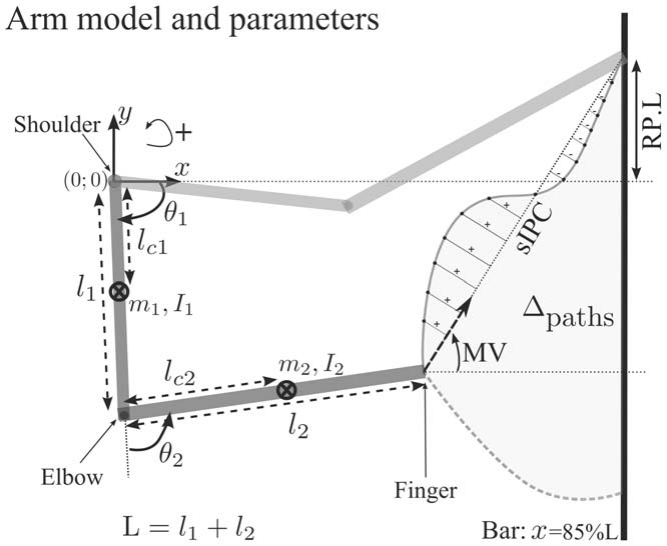
Model of the arm and definition of the parameters. The extrinsic and intrinsic coordinates are denoted by 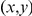 and 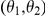, respectively. 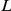 is the total arm length, while 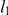 and 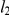 are the upper arm and forearm lengths. The subscript 1 denotes the shoulder joint. These segments have mass 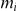, inertia 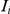 and distance to the center of mass 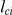, with 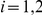. The Cartesian bar equation is given by 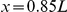. The solid and dotted lines are the measured and simulated paths, respectively. The parameters RP, MV and sIPC are the reached point, movement vector angle and the signed index of path curvature.

The shoulder joint was defined as the origin of the frame of reference (see Figure 3).

Finally, additional task-relevant parameters were computed. The *endpoint position consistency index* (CI) was defined as the ratio between the standard deviation of the fingertip position on the 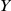-axis and the length of the reachable region. This set was computed from the intersection points between the bar and a shoulder-centred circle of radius 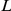. The CI parameter provides information concerning the percentage of the bar used by the subjects. The smaller is this index, the more consistent was the subject's behavior for the selection of a terminal point on the bar. The location of the reached point was calculated with respect to the shoulder position and normalized by the subject's arm length 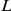 (referred to as RP). In other words, the location of the endpoint on the bar is 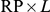 (in meters). In order to detect whether subjects chose to move upward or downward, we computed the *movement vector* angle (denoted by MV) defined as the counterclockwise-oriented angle between a horizontal line and the line connecting the initial and terminal fingertip positions.

Moreover, to assess whether the finger path had a convex or concave curvature, we computed the signed Index of Path Curvature (sIPC). This was defined as the averaged ratio between the maximum path deviation from a segment connecting the initial-final finger positions and the length of this segment, attributing a positive sign when the finger position was above the straight line (for concavity). Thus, this parameter evaluates the average or global convexity or concavity of a hand path. In addition, joint coupling was calculated as the determination coefficients between the shoulder and elbow angular displacements. In order to compare models predictions and measured data, we computed the *area between paths*. Given the complexity of the polygon to be integrated (whose area is denoted by 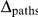), we used a numerical method based on the evaluation of the integral with a random sampling of the integration region (the standard Monte Carlo integration method). Note that, throughout this paper, we will distinguish the terms *path* and *trajectory* in that the former refers only to the graph of the trajectory (i.e., the trajectory also includes the time-course).

### Statistical analysis

We used quantile-quantile plots to visually check that the data were normally distributed (*qqplot* Matlab function). Shapiro-Wilk's test was used to quantify these observations for some relevant parameters. One-way ANOVAs were also performed to analyze the effects of the initial posture on certain parameters. Post-hoc tests were conducted with Scheffé's test when necessary and appropriate (the chosen threshold was 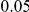).

### Modeling

Previous models of optimal control for arm movements were originally designed by their respective authors on the basis of some particular assumptions and restrictions. In order to compare several different costs proposed in the literature and to apply the inverse optimal control technique described thereafter, we consider a homogeneous framework, compatible with most existing models. The next subsections describe the musculoskeletal model, the inverse and direct optimal control techniques that we employed. Details are deferred to the supplementary documents Text S1 and Text S2.

#### Model of the musculoskeletal system

It will be shown that the recorded 3-D arm movements approximately lied on the para-sagittal plane. Thus, a reasonable approximation for modeling is to consider the arm as a two-joint rigid body moving in the vertical plane. A classical application of Lagrangian mechanics allows us to express the arm dynamics using the general form [23]:
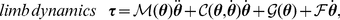
(2)where the variables 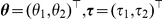 denote the joint angle and torque vectors, respectively. A *dot* above a variable stands for the time derivative. The quantities 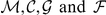 are the inertia matrix, the Coriolis/centripetal terms, the gravitational vector and the viscosity matrix, respectively. The explicit expressions of the above quantities and numerical values are provided in the Text S1 (Section 1).

Furthermore, we modeled the fact that the joint torques 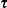 are smoothly generated by muscle contractions, a phenomenon which is subject to a certain dynamics:
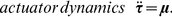
(3)


The control variable 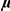 is the motor command and can be thought as the neural input to muscles given by motor neurons. For compatibility between models and simplicity, we thus assume that the effect of muscle contraction is mechanical and that motor neurons control directly the acceleration of torques. From now on, we will denote by 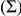 the system composed of Equations 2 and 3. Some constraints on the state and control variables were also taken into account for biological plausibility (see Text S1).

It is noteworthy that, for considering several costs within the same framework, we did not model neither agonist and antagonist muscles, nor the complex mechanism of muscle contraction. Nevertheless, additional verifications (through direct optimal control) suggested that the results presented in this study do not critically depend on this choice (see Text S2, Section 1). For instance, modeling agonist/antagonist muscles as second order low-pass filters [24] does not improve drastically the predictions of the effort model, which is the most sensitive model to the actuator dynamics. Very small changes in the predicted trajectories were obtained for the other costs. The main reason is that the optimal trajectories were found quite robust with respect to changes of the actuator dynamics (up to some extent of course; for instance, when the muscle dynamics allowed moving the arm along identical paths).

#### Inverse optimal control

The goal of inverse optimal control is to automatically infer the cost function from observed trajectories that are assumed to be optimal. Thus, in inverse optimal control problems (inverse OCPs), the optimal solution is known and the objective is to recover the performance criterion which has been optimized. Addressing the motor planning problem in this way is generally more difficult than using the more standard direct optimal control approach, which consists of guessing a plausible cost and comparing its predictions with the experimental data. However, inverse OCP is better suited to provide, with less a priori, the cost or mix of costs that must be optimized to replicate the measured arm trajectories. In this paragraph, we present a numerical method for solving an inverse OCP, which was initially described by [25] and successfully applied to path planning during locomotion in humanoid robotics.

The method relies on the selection of a set of plausible costs. For the optimal control of arm movements, several costs were already proposed in the literature. The models generally fall into four general classes, each of which making different assumptions on the relevant variables for the CNS. First, there are the *kinematic models*: the minimum hand jerk [11], the minimum angle jerk [26], or the minimum angle acceleration with constraints [27]. They suggest a maximum of smoothness in either the Cartesian or joint spaces. Then inverse kinematics and/or inverse dynamics are required to obtain the actual control 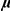. Those models belong to the family of Minimum Squared Derivatives (MSD) costs. Throughout the paper, we shall use the generic term *smoothness* in the broad sense of “having small high-order derivatives” [28]. In particular, the integrated squared acceleration and integrated squared jerk are just members of the class of MSD costs, which favor motion smoothness to different degrees in joint coordinates [29]. Alternatively, *dynamic models* were proposed to avoid these inverse dynamics computations, such as the minimum torque [9] or the minimum torque change models [12], [30]. At the junction of kinematic and dynamic models, the *geodesic model* suggests that the brain may select the shortest path in configuration space with respect to the kinetic energy metric [31]. This model is called geodesic due to the fact that it seeks shortest paths in a Riemannian manifold. *Energetic models* were also considered in several studies, in particular those involving the minimization of work of torques (see [32] for the peak of work, [33] for the positive work, and [34] for the total absolute work). Here, we will only consider the total absolute work because this corresponds to the mechanical energy actually spent to move the arm. Finally *neural models*, often referred to as minimum *effort* models, were designed to optimize the amount of motor neurons activity during a movement [35], [36]. Although other models for movement planning were proposed in the literature, they could not be integrated to the present work for one of the following reasons: (1) they fall in the stochastic context, (2) they require an accurate modeling of agonist/antagonist muscle mechanisms or (3) they do not assume optimal control at all. Indeed, a limit of the present methodology is to be able to describe models within a single mathematical framework, defined by Equations 2–3 and the specification of a cost function (see Table 1).

**Table 1:** Classical cost functions already proposed in the literature.

Criterion	Cost function ( 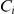 )	References
Hand jerk	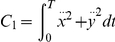	[11]
Angle jerk	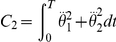	[26]
Angle acceleration	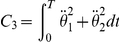	[27]
Torque change	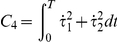	[12], [30]
Torque	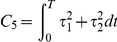	[9]
Geodesic	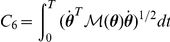	[31]
Energy	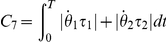	[33], [34]
Effort	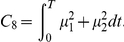	[35], [36]

Classical cost functions already proposed in the literature and that are used in the present study. Some of them were not originally formulated as OCPs, but for the purpose of this paper, they were reformulated in this framework.

Therefore, we selected the following costs for further investigation: hand smoothness (Cartesian jerk), joint smoothness (angular acceleration and angle jerk models), torque change, torque, geodesic, mechanical energy, and neural effort (each of which denoted by 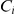, 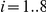, see Table 1 for details). From these eight biologically plausible costs, we could build other costs (called *hybrid* or *composite* and denoted by 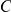), expressed as a weighted linear combination:
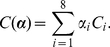
(4)


The parameter 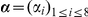 is referred to as the weighting vector, whose elements are non-negative. A weight of zero means that the corresponding cost does not contribute to movement planning.

Thus, the OCP corresponding to the cost 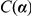 can be stated as follows: *Find a control* 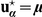 *and the corresponding trajectory* 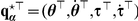 *of system* 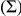 *, connecting a source point* 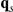 *to a final point on the target manifold* 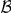 *in time* 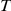 *and yielding a minimal value of the cost* 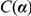.

Let us denote this problem by 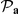. Here, the target is the vertical bar, given by the equation 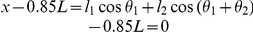 in Cartesian and joint coordinates, respectively. Since subjects had to reach the bar with zero velocity and zero acceleration, the manifold could be written in state-space as 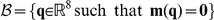, for some vector-valued mapping 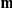 (see Text S1, Section 2.1). The fact that this mapping is surjective is exactly the reason why the task is redundant, even though we modeled the arm as a simple two-joint arm moving in a plane.

Let us now denote by 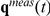 the measured/experimental trajectory in state-space. Then, the core of the inverse optimal method is to formulate the so-called “bi-level” problem [25]:
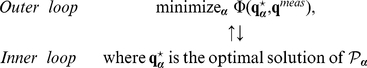
(5)


The outer loop (also referred to as “upper level” by some authors) consists in solving an optimization problem for the unknown 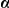 in order to find the best match between the optimal trajectory 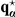 and the measured trajectory (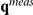). The inner loop (“lower level”) precisely consists in computing the optimal trajectory 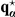 corresponding to the current cost combination 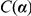 (for this step, see the next subsection). It is often desirable to generalize the above bi-level problem to the case where several experimental observations are available (i.e. several 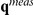). This allows better characterizing the cost function: roughly speaking, the more the observations, the more relevant the fitting. In such a case, several direct OCPs have to be solved during the inner loop and the metric used in the outer loop simply rewrites as a sum over all those observations. In this study, we used the five starting postures (P1 to P5) as observations to identify a unique cost reproducing at best the behavior of a subject.

How to define a good “metric” in the space of trajectories is still an open question in motor control [17]. Depending on what movement features are considered to be important, various functions 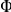 can be constructed. This choice is however crucial for the inverse optimal control results since it quantifies how well a given model replicates the experimental data. In this paper, we consider two possibilities. The first metric (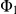) is based on measuring the Cartesian and curvature errors of a simulated trajectory with respect to a reference trajectory (here the average experimental trajectory). The second metric (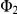) relies on a statistical model of the recorded trajectories (encoded in a Gaussian Mixture Model, see [37], [38]) and likelihood estimations of an optimal trajectory given that model. The advantage of this metric is that it takes into account the inter-trial variability of the human behavior by penalizing model/data mismatches only for the features that are clearly defined by the experimental trajectories. In the following, only the results obtained with the first metric are analyzed in depth, but our conclusions still hold when using the second metric. All the details can be found in the Text S2 (Section 4.1).

Solving the bi-level problem is not straightforward for several reasons. First, the objective function 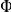 in the outer loop is quite long to evaluate because a direct OCP must be solved before (this can take a few minutes for one evaluation). Moreover, it might be relatively noisy because only an approximation of the optimal trajectories can be obtained so that 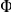 can be non-differentiable with respect to 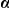. Consequently, the minimization problem of the outer loop had to be solved with a robust derivative-free technique. Here, we used the method developed by [39] which is an extension of the state-of-art Powell's method based on local quadratical approximations of 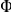 [40]. This method is called CONDOR for COnstrained, Non-linear, Direct, parallel optimization using trust region method for high computing load function. It was found to be more efficient than standard pattern search and stochastic-based (e.g., genetic algorithms) methods for the present purpose. This observation is in agreement with [25] who used similar numerical techniques for inverse optimal control.

To improve the algorithm efficiency, we found useful to appropriately scale the step size along each dimension of the search space. We used a re-scaling vector, 
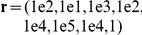
, obtained from multiple simulations of point-to-point movements using single-cost criteria to evaluate the magnitude of the optimal movement costs. This re-scaling/normalization is generally meaningful because different costs have different units. We could have avoided using this re-scaling, but it turned out to speed up the inverse optimal control procedure and to yield better local optima. Another method to set this re-scaling vector is presented in the Text S2 (Section 4.2) and is based on sensitivity analysis, i.e. on the evaluation of the gradient of the optimal cost 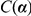 at a point 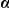. Both methods turn out to yield pretty similar results. It has to be also underlined that among the eight elements of 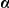, only seven were actually independent. Indeed, note that the OCPs corresponding to the costs 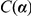 and 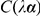 with 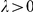 are identical. As in [25], the practical strategy for this was to fix one component of 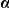 equal to one and to adjust the remaining components. Whenever this choice turned out to be inappropriate, this was apparent during the optimization process. In that case, this component should be set to zero and, then, another one should be set to one. Setting the angular acceleration coefficient to one resulted to be a good choice in this study. To test robustness of the procedure, it was initialized with random non-negative values or directly with the vector 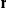 to initially give a similar weight to all the costs. Moreover it was run for all the subjects in order to verify the consistency of the findings. The algorithm always converged in a few hundred of iterations to a (local) minimum of 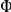 and the resulting cost combination vector (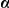) was found quite stable with respect to the initial guess. Applying the inverse optimal control algorithm to all the subjects required to solve roughly 10 000 direct OCPs (about 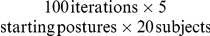).

#### Direct optimal control

As explained above, the inner loop of the bi-level problem requires solving direct OCPs for a given 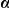. This is also a computational problem *per se*, especially when dealing with complex costs and dynamics. However, in the deterministic context that we consider here, there exist efficient numerical techniques to find approximate solutions. A classical method is to transform the OCP into a nonlinear programming (NLP) problem with constraints. Here we used an orthogonal collocation method, precisely the Gauss pseudospectral method. This method is efficiently implemented in the open-source Matlab software 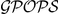 [41]–[43]. The NLP problem was solved by means of the well-established numerical software SNOPT [44]. This pseudospectral method relies on time discretization at some points chosen to be the Legendre-Gauss ones, i.e. the roots of a certain order Legendre polynomial. Then, the state and control are approximated using interpolating Lagrange polynomials. This method was proven to be very efficient for a large class of OCPs. Our own tests confirmed that the software performed very well for the costs proposed in the motor control literature and was even consistent when the optimal solution involved discontinuous optimal controls. This verification was performed using a second method for solving an OCP, relying on the direct application of Pontryagin's maximum principle (PMP, [45]). The PMP provides necessary conditions of optimality and can allow obtaining very precise solutions. After some analytical calculations, the PMP generally leads to a boundary value problem that can be tackled by a shooting method. However, in practice, a shooting problem is also a difficult computational challenge because the radius of convergence may be quite small and, therefore, a good initial guess of the optimal solution is usually required to get robust convergence. Therefore, a standard approach is to initialize the shooting method by using a guess arising from a numerical optimal control technique. Interestingly, the PMP can also deal with point-to-manifold problems by adding transversality conditions on the terminal costate vector so that its use was purposeful in the present study. Using this approach, we thus verified that the numerical method provided good approximations of the optimal trajectories, which was an important step for the success of inverse optimal control. Details and instances of resolution using the PMP are provided in the Text S1 (Section 2.2).

#### Models versus experimental data comparisons

Apart from the inverse approach, a verification was also conducted by directly analyzing the predictions of each single cost model (defined by 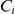). To this aim, we simulated every movement recorded in the experiment. Precisely, we simulated the original protocol for the 20 subjects, assuming that they plan their movements by minimizing one of the costs under investigation. Therefore, anthropometric parameters were set to realistic values for each participant (see Table S1 in Text S1). Interestingly, this also allowed testing the sensitivity of models with respect to parameters such as inertia, mass and segment lengths. A number of initial parameters were set from experimental measures, namely the movement duration 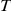, the initial arm posture 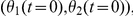 and the horizontal bar-shoulder distances to better match the initial experimental conditions. In total, we ran 16 000 simulations (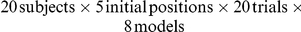) and used their predictions for subsequent analyses. These simulated data were then treated using the methods described in Materials and Methods (Motion Analysis subsection). We eventually estimated the sensitivity of the optimal cost with respect to the endpoint selected on the bar in order to evaluate the consequence of sub-optimality on the final point. To this aim, movement costs were evaluated by solving an direct OCP for every possible final finger point and every model. The reachable region on the bar was discretized every 3 cm (i.e., this region was subdivided in 30 segments) and the optimal cost for each point-to-point movement was computed.

## Results

### Experimental observations

#### Task achievement and general movement features

The behavior of a representative subject is illustrated in Figure 2B. Participants were generally quite precise in executing the movement. The horizontal constant error (distance to the bar on the 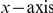) was 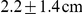 on average across subjects and initial positions, indicating that the subjects controlled their movements quite accurately in the antero-posterior axis. The variable error (i.e., the endpoint dispersion) was 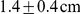. The lateral error was disregarded here because participants approximately displaced their arm in a vertical plane. Indeed, principal component analyses performed on the 3-D coordinates of the moving markers for each subject showed that the variance accounted for by the two first components was more than 98% and that the angle between normal vectors of this plane and the vertical plane defined by the acquisition system was about 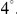 Therefore, movements could be considered as approximately effected in a vertical plane and subsequent analyses could be performed on the projected data without a large loss of information.

Table 2 reports the general motion features. Movement duration slightly varied across participants and starting positions, and lasted about 700 ms in general (ANOVA, 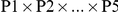, 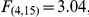 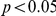). The distance covered by the hand significantly depended on the initial posture (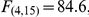 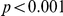), and therefore, the average speed varied accordingly. In particular, the smaller curvilinear distance was obtained when starting from P2 (about 30 cm) and the larger one from P4 (about 70 cm).

**Table 2:** General movement features.

	P1	P2	P3	P4	P5
Movement duration (s)	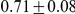	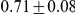	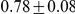	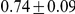	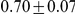
Mean velocity (m/s)	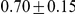	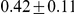	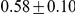	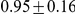	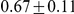
Time to Peak velocity	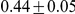	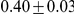	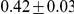	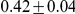	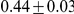
Vpeak/Vmean	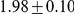	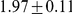	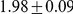	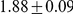	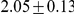
Curvilinear distance (m)	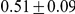	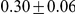	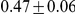	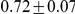	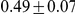
Constant error on 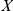 -axis (m)	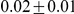	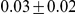	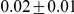	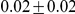	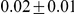

Means and standard deviations are reported across subjects and starting postures.

#### Inter-trial consistency of the behavior

**Figure 4:**
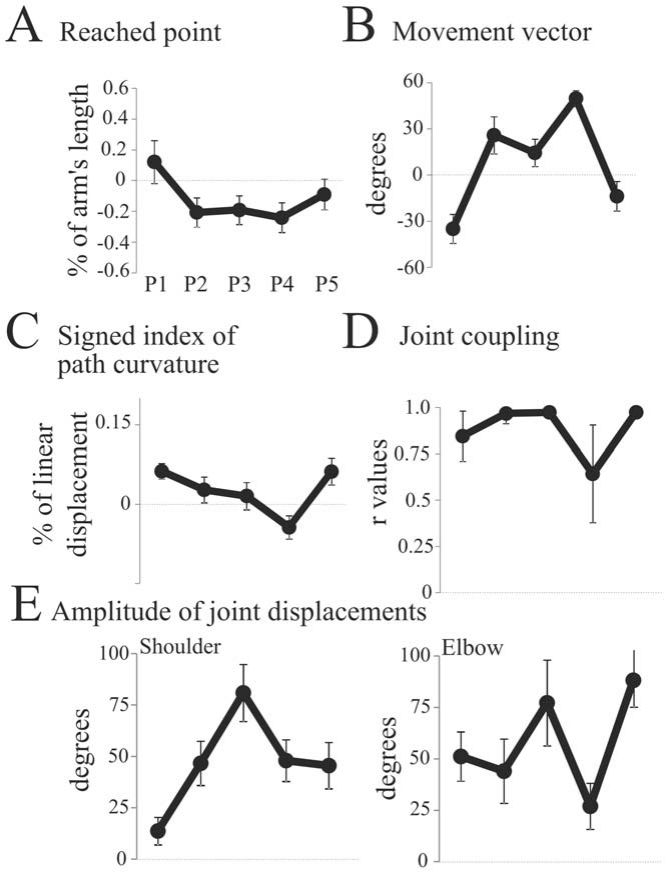
Quantitative experimental results. A. *Reached point* (final finger position) on the bar for each initial posture from P1 to P5 (RP parameter). The unit on the vertical bar is normalized by the arm length (percentage). The horizontal zero baseline is the level of the shoulder joint. Each point indicates the average location of the pointing movement, and error bars indicate the variability (standard deviation) across subjects. B. *Movement vector* angle (MV). The graph gives the angle between the movement vector and the horizontal line. Negative and positive values correspond to downward and upward movements, respectively. C. *signed Index of Path Curvature*: The graph depicts sIPC values for every initial posture. Positive and negative values correspond to globally concave and convex paths, respectively. D. Joint coupling. 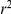 values are reported. Low values indicate low level of correlation between the shoulder and elbow angular displacements. E. *Amplitudes of angular displacements*. The graphs correspond to the shoulder (left) and elbow (right) joints, respectively. The magnitude of joint displacements (in degrees) is given for all initial postures.

Subjects could reach wherever they desired on the bar (i.e. on the vertical axis). Therefore, it appeared important to verify whether their behavior was consistent or not. An analysis of the consistency index (CI, a parameter similar to a normalized *variable error* along the vertical axis, but this is not an error in this task!) showed that the subjects used only 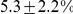 of the reachable region on the bar (average across subjects and conditions). In terms of absolute measure this corresponded to a standard deviation of 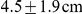 on the vertical axis. In other words, it was three times larger than the variability measured on the antero-posterior axis. Thus, rather than using all the available freedom across trials, participants reached toward preferred regions of the bar. These regions are depicted in Figure 4A. In particular, such a inter-trial consistency was present whatever the initial posture without significant differences (ANOVA, 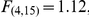 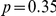).

It has to be noted that among all the tested subjects, only two behaved quite atypically. One of them exhibited a highly variable behavior, exploring the whole bar across trials. The second one started to increase drastically his trial-to-trial variability during the second half of the experiment while being invariant in the first half. This kind of behavior can be considered as quite marginal since it appeared for only 2 of our 20 participants, and reflected uncommon motivations/intentions.

#### Endpoint on the bar

The average behavior is illustrated in Figure 2B. A qualitative analysis of the RP parameter showed that the endpoint depends on the initial posture of the arm. A statistical analysis revealed that this effect was significant (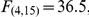 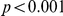). Post-hoc analysis showed that the terminal point when starting from P1 was significantly different from all the others (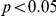). Similarly, the point reached when subjects started from P5 was significantly different from all the others. Finally, no significant difference was found within the group P2–P3–P4, although a trend was apparent and robust across subjects. Figure 4A summarizes these observations and also depicts the location of the terminal point for each posture with respect to the shoulder and eye levels (evaluated through Frankfurt plane).

Finally, we also conducted an analysis on the movement vector angle (see Figure 4B). An ANOVA revealed a significant effect of the starting posture on the MV parameter (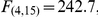 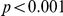). The MV values were negative for P1 and P5 indicating that the hand moved downward. The most vertical movements were obtained when starting from P1 and P4 (average MV equal to −35 and 50 degrees, respectively). Movements starting from P2, P3, and P5 were the most horizontal (MV values about 25, 15, −14 degrees, respectively).

#### Shape of the finger paths

A visual inspection of the shape of paths showed that they were generally curved in the 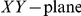. It is visible in Figure 2B that the fingertip paths have typical curvatures and that they strongly differed from straightness. An analysis of the signed index of path curvature parameter (sIPC) shows that this result was quite robust across subjects (see Figure 4C). For most initial postures, paths were globally concave, except for P4 for which the fingertip path was clearly convex. An ANOVA confirmed these differences since a significant effect of the starting posture on the sIPC parameter was found (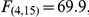 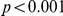). Post-hoc tests revealed that three distinct groups could be extracted. The convex group (P4), the very concave group (P1, P5), and the slightly concave group (P2, P3). It is noticeable that for the latter group, some subjects indeed produced quasi-straight paths (5/20 for P2 and 10/20 subjects for P3). Nevertheless, we never measured significantly convex paths when starting from P2 and P3. Overall, the index of path curvature was a quite invariant movement feature.

#### Time-course of joint and finger trajectories

Angular displacements were generally monotonic for all subjects and conditions, except for instance for posture P4 at the elbow joint (see Figure 2C first column for the typical subject). Through correlation analyses, we determined that the forearm and upper-arm segments were globally well coupled. The determination coefficient between the elbow and shoulder angles was high on average (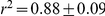). However, the starting posture had a significant effect on the joint coupling (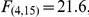 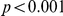). A post-hoc analysis showed that P1 and P4 were significantly different from other initial postures. Movements starting from P4 showed a reduction of joint coupling for 13/20 subjects (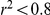) and, more generally, the determination coefficient decreased for all subjects compared to initial postures P2, P3 or P5. The results were similar for P1, for which the 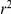 value decreased significantly for the 20 participants (see Figure 4D). The low joint coupling measured in conditions P1 and P4 were linked to the non-monotonic nature of the angular displacements and, likely, to the relatively small amplitude measured at the shoulder and elbow joints, respectively (about 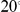 on average, see Figure 4E). In fact, an analysis of the angular displacements magnitude showed (Figure 4E) that movements starting from P1 mainly involved an elbow rotation with a small rotation at the shoulder joint. Starting from posture P2 or P3 involved similar angular excursions at both joints, while from posture P4, subjects tended to mainly rotate the shoulder joint with a significantly smaller forearm flexion. Finally, movements from posture P5 implied large rotations of both joints (but twice larger for the elbow).

The finger velocity profiles were always bell-shaped, meaning that movements were one-shot without terminal adjustments (that is they showed unique acceleration and deceleration phases, as depicted in Figure 2C second column for the typical subject). Velocity profiles presented some asymmetry: acceleration always lasted less than deceleration, whatever the starting position. Table 2 shows that, on average, acceleration represented only 42% of the whole movement time (TPV parameter). The ratio Vmean/Vpeak ranged between 1.8 and 2.1 (mean 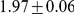), indicating quite narrow velocity profiles in general (for comparison, the value predicted by the minimum hand jerk model is 1.875).

### Cost identification

#### Inverse optimal control results

**Figure 5:**
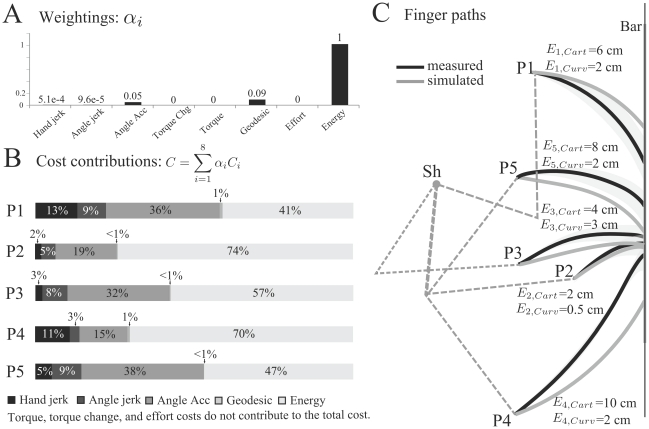
Inverse optimal control results: details for the most typical subject. A. Weighting coefficients, i.e., elements of the vector 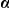 (normalized by the maximum value). B. Contribution of each cost ingredient with respect to the total cost, for each simulation. The contribution of the 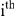 cost is computed as 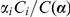. It is visible that mainly the energy and the angle acceleration are involved in general, with low contributions of the hand and angle jerks and a residual contribution of the geodesic cost. Torque, torque change, and effort costs do not contribute at all. C. Finger paths obtained from the best cost combination found by the inverse optimal procedure. Errors between the measured paths and the simulated ones (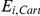 and 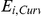 parameters) are reported, for each initial posture. Note that this is the best criterion, and that any other cost combination would replicate the data less accurately with respect to metric 1.

By means of inverse optimal control, we could identify the cost or mix of cost that best accounted for the experimental data. Figure 5 depicts the results of the method applied to the most typical subject, previously presented in Figure 2B. For this subject (referred to as S1), the algorithm converged to a particular hybrid cost, defined by the weighting vector shown in Figure 5A (using metric 1). This vector was composed of energy, geodesic, angle acceleration, hand jerk and angle jerk (given in decreasing order of weights). Other variables such as torque, torque change and effort had a weight exactly equal to zero (the lower bound was thus reached by the algorithm). However, the weighting vector does not directly reflect the contribution of each element to the total movement cost. For instance, for this subject, the total optimal cost was mainly composed of energy (on average 58% of the total cost) and angle acceleration (on average 28%), as illustrated in Figure 5B. Although the geodesic element had a non-negligible weight, its contribution was less than 1% in general. It is also apparent that the contribution of each cost depends on the starting position. Nevertheless, in general, relatively small contributions of angle jerk and hand jerk were found. The minimization of angle acceleration and angle jerk both aim at maximizing the joint-level smoothness. Taking this into account, the joint smoothness contribution to the total cost can be increased to 35% for this subject. Figure 5C illustrates the trajectories predicted by this particular combination of the 8 elementary costs. Despite the task redundancy and the simplifications made in modeling, this hybrid model captured quite well the location of the endpoint on the bar and the convexity/concavity of the finger paths. The maximal distance between the simulated and actual paths was 6 cm on average while the maximal difference between the simulated and actual path curvatures was about 2 cm on average (the average errors are obviously smaller). Table 3 reports the fitting errors for all subjects, the typical subject being denoted by S1.

**Table 3:** Reconstruction errors after inverse optimal control.

Subject	S1	S2	S3	S4	S5	S6	S7	S8	S9	S10
Mean error (cm)	4.1	3.9	1.8	6.5	3.3	3.5	4.6	3.9	3.4	2.9
Subject	S11	S12	S13	S14	S15	S16	S17	S18	S19	S20
Mean error (cm)	3.2	6.1	2.5	4.6	2.5	1.7	2.7	6.2	3.6	1.8

Inverse optimal control fitting errors using metric 1. For each subject, the error value is averaged across all starting postures and, more precisely, it is computed as 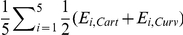 (see Text S2, Section 4).

**Figure 6:**
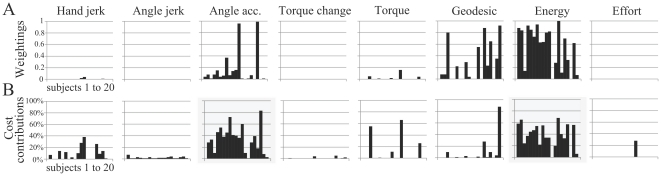
Inverse optimal control results for the 20 subjects using metric 1. A. Weighting coefficients, i.e. elements of the vector 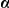 (normalized such that the sum equals 1). Each bar corresponds to one subject. B. Contribution of each cost ingredient to the total cost, for each subject. The energy and angle acceleration costs, which are predominant in the total movement cost, are highlighted with shaded areas. This result is not evident when looking only at the weighting vector.

Similar results were obtained for several subjects, despite the differences in their movement durations and anthropometric parameters. The best-fitting weighting vectors 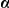 constantly showed the presence of mechanical energy expenditure (absolute work of torques, 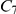) and joint smoothness terms (angle acceleration/jerk energy, 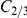), while other terms appeared more sporadically (Figure 6A). Nevertheless, due to the different magnitudes of the cost ingredients, analyzing their relative contribution to the total cost revealed itself insightful (Figure 6B). Particularly, energy and joint smoothness turned out to be consistently present in the optimal composite cost (about 40% and 35%, respectively, on average). Thus, their cumulative contribution represented the main part of the total movement cost. Some contributions of the hand jerk (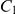), the geodesic (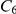) and the torque (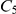) costs were also found (about 8% on average). Nevertheless, these values were relatively small and erratically present in the total cost so that they might be considered marginal. The effort and torque change costs (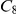 and 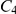) almost did not contribute to the total cost and, thus, did not seem to be optimized in this task. Although not shown here, when restricting the inverse optimal method to initial postures P2 and P3, it was found that the mechanical energy had to be involved in the cost, otherwise the concave curvature of the finger paths could not be reproduced. Also, a meticulous inspection of Figure 6B shows that two subjects did not minimize the mechanical energy expenditure at all. For them (subjects S12 and S18), the fitting error was significantly larger than for the other subjects (6.1 and 6.2 cm respectively, see Table 3). It is worth noting that these two subjects corresponded to the ones who exhibited an atypical behavior, characterized by a very large variability during the experiment. This finding is interesting since moving arbitrarily to different points on the bar is obviously non-optimal with respect to the energy expenditure. Although we restricted the inverse control to the average behavior of subjects, it turned out that the inverse method could nevertheless detect that these behaviors were not optimizing the same cost. A couple of subjects also presented slightly different cost contributions, without excluding nevertheless energy and smoothness terms.

Taken together, the above results provide clues on which costs must be considered to capture the basic characteristics of human movements during the reaching-to-a-bar task. The majority of subjects (15/20) clearly adopted a behavior optimizing a well-characterized hybrid cost, essentially mixing the absolute work and the angular acceleration (i.e., the other costs are somehow residual). Consequently, for the further investigations using direct optimal control, we included this identified composite cost to compare it with the basis costs. Since the ratio between the weighting coefficients of energy and angle acceleration was roughly 10∶1, the hybrid cost was chosen to be 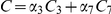 with 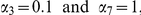 the other coefficients being set to zero. From now on, this weighting vector will be kept constant for all conditions and all subjects to avoid overfitting and unfair comparisons between models.

#### Direct optimal control verification and comparison

**Figure 7:**
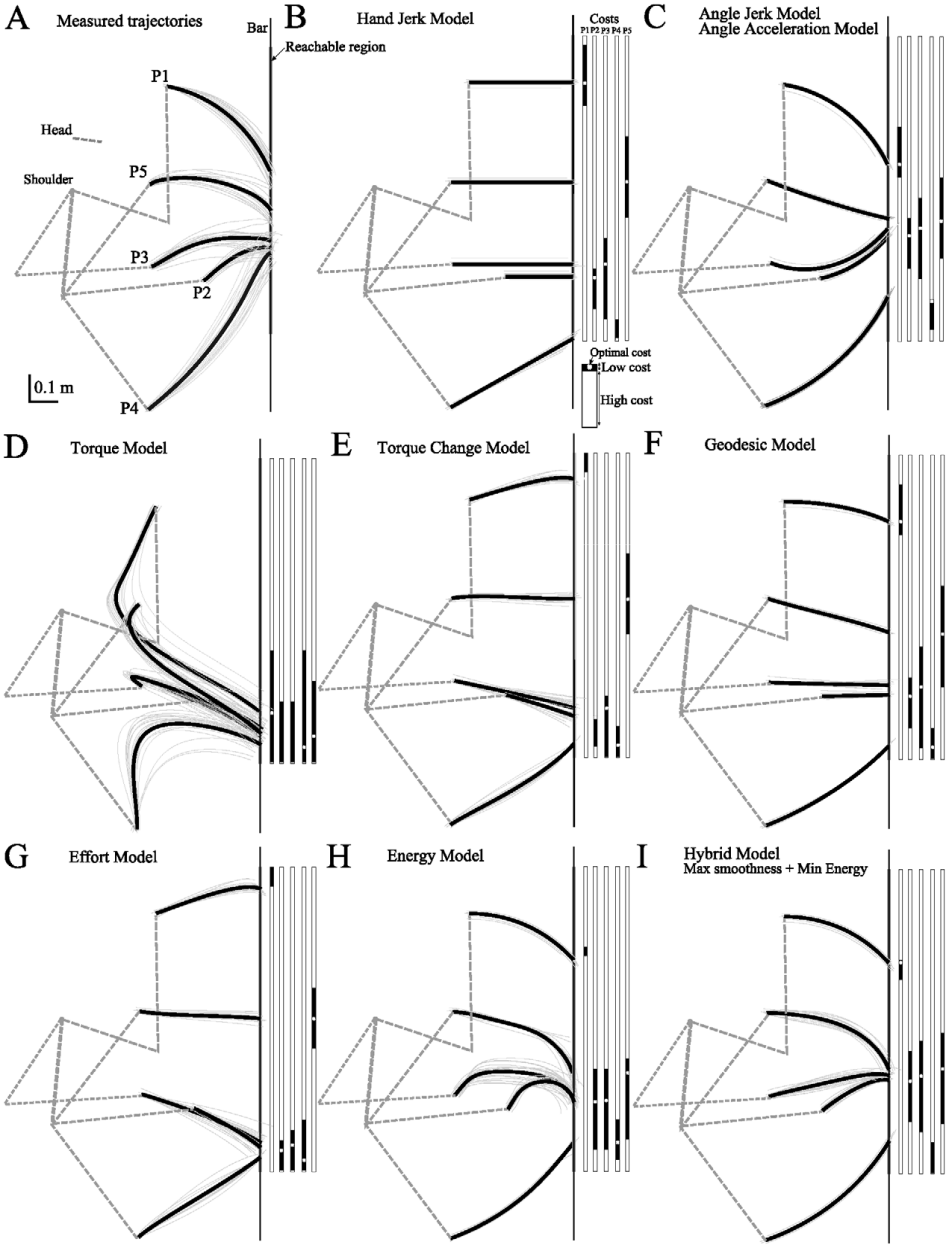
Predictions of the different tested models. A. Typical experimental data in order to facilitate comparisons (already depicted in Figure 2). B–H. Predicted hand paths for each model. I. Hybrid model, maximizing smoothness and minimizing energy (with a ratio 10∶1 for the energy component). Black and white bars are reported to show the regions on the bar for which the cost is relatively close to the optimal one (here, black areas correspond to movement costs below the 10% threshold relative to the minimum cost value).

A preliminary inspection of Figure 7B-I shows that models predicted highly different trajectories. This was expected because of the large freedom given by the bar reaching experimental paradigm and the results introduced in Figure 1. A quick overview on these results suggests that the hybrid model performed better than all the other single-cost criteria. Qualitatively some models yielded geometric paths that were clearly incompatible with the typical experimental data that we have reported again in Figure 7A to facilitate comparisons.

**Figure 8:**
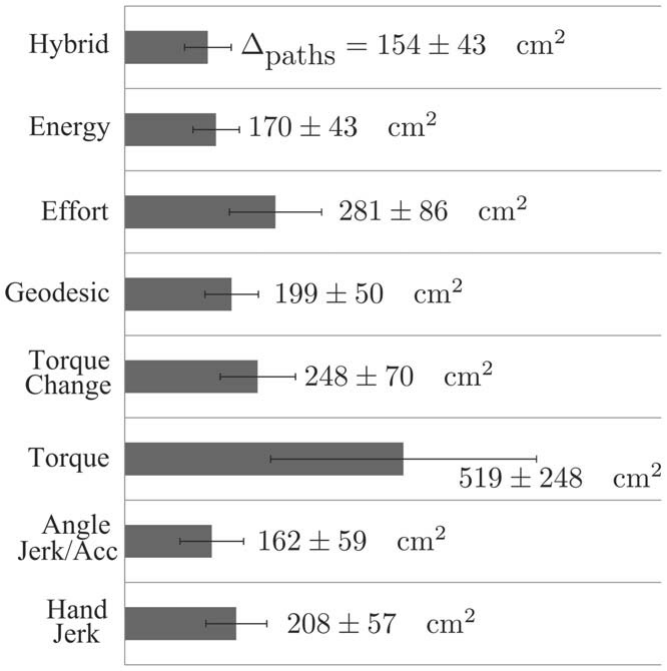
Areas between simulated and recorded finger paths. This parameter qualifies as a general error measure. Values were first averaged across initial postures for each participant, and then, the mean and standard deviation were finally reported across participants. It is apparent that the energy and angle jerk/acceleration models performed quite well (with a lower standard deviation for the energy model), while the geodesic and hand jerk models performed moderately. The worst models were the torque change, effort and torque models, given in decreasing order of performance. The best model was the hybrid model, in agreement with the results provided by the inverse optimal control approach.

To quantify the matching between models and real data an analysis of the finger path was conducted, including all subjects and all initial postures. The difference between simulated and measured paths was first measured through the area 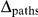 (see Figure 8). It is apparent that, on average, the best single models were the minimum angle jerk/acceleration (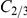) and minimum energy (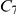) models, while the minimum torque (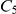) predicted non-realistic paths and resulted in very large errors. The minimum torque change (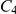) and minimum effort (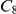) models also performed quite poorly, while the geodesic (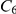) and minimum hand jerk (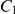) had a moderate level of fitting. The hybrid model (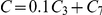) replicated globally better the experimental data, in agreement with what was suggested by the inverse optimal control approach. Note that in this analysis a fixed composite cost was used even though the inverse results suggest that the actual weighting may be subject-dependent.

**Figure 9:**
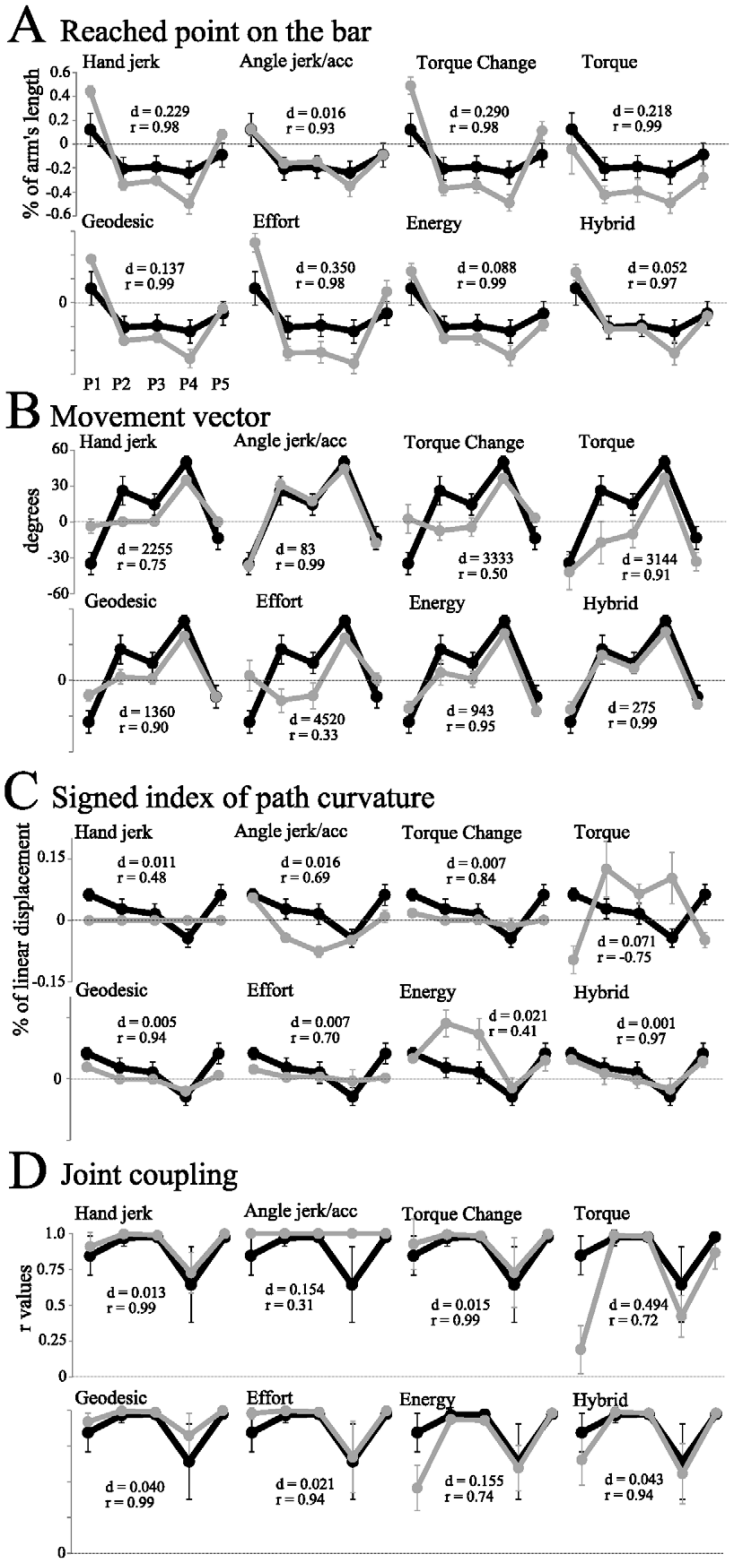
Comparisons between models and real data, for relevant parameters. A and B depict the reached point (RP) and movement vector (MV) parameters, which are the relevant parameters for the finger path. An analysis confirms that energy and angle jerk models, as well as the hybrid model, are quite efficient in predicting the terminal point on the bar and the movement direction (upward or downward). C and D depict the signed index of path curvature (sIPC) and joint coupling (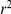), and are reported for the sake of completeness. However, they are not relevant when the final point is poorly predicted by a model. It is apparent that only the hybrid model is able to predict successfully these additional parameters (sIPC and joint coupling 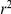). Parameters reported on the graphics: parameter 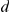 is the cumulative error across all starting positions 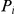: 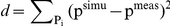, with 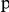 being one of the following parameters: RP, MV, sIPC, or joint coupling; parameter 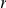 is the correlation coefficient between the simulated and measured data.

A specific analysis of task-relevant parameters was also performed (see Figure 9). The most basic task parameter was the relative reached point on the bar (RP, Figure 9A). The angle jerk/acceleration models predicted remarkably well where subjects did point on the bar on average, with a mean error of approximately 6% of the arm's length, i.e., about 5 cm. The second model was the energy model which predicted the final finger position with about 11 cm of error on average. The hybrid model performance was intermediate (about 8 cm), which was still reasonable with respect to the standard deviation exhibited by subjects in general. Other models tended to make large errors on the location of the point reached on the bar (up to 23 cm for the effort model, i.e. a cumulative error 22 times larger than for the best model). This was confirmed by an analysis of the movement vector angle, reflecting the pointing direction (Figure 9B). Only the minimum angle jerk/acceleration models and the hybrid model replicated well the sequence downward-upward-upward-upward-downward for initial postures P1–P2–P3–P4–P5 (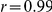 with an error of 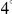 on average for MV). The minimum energy was also relatively efficient in capturing this sequence (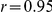 with an error of 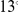 on average for MV). Again the most discrepant model was the minimum effort model with more than 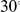 of error on average (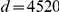) and a behavior across initial postures poorly reproduced (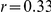). Above all, it appeared that where to reach the bar was best explained by angle jerk/acceleration, energy or a combination of them (hybrid model).

Concerning the shape of the path (sIPC parameter, Figure 9C), the sequence concave-concave-concave-convex-concave (following the five postures) was not well predicted by the angle jerk/acceleration models (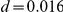, 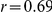). In particular for P2 and P3, these models predicted strongly convex paths to reach the bar, while concave paths were observed experimentally. In fact, all single models almost predicted the same shape, except the torque, energy and hybrid models which predicted concave paths. Since the torque model was very discrepant with the data in general and since the energy model clearly overestimated the concavity of the paths for P2 and P3, only the hybrid model predicted well the paths curvature (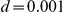 and 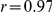). Interestingly, this model relies on two extremes: the angle acceleration predicted very convex paths while the energy model predicted very concave paths. Finally, note that the geodesic model was reasonably accurate to reproduce the quasi-straight paths produced by some subjects when starting from P2/P3 (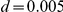 and 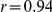) and the final point on the bar, so that this model performed relatively well in general. The same cannot be concluded for the effort or torque change models because these models were particularly inefficient in predicting the final finger position (Figure 9A and 9C).

The joint coupling analysis (Figure 9D) revealed that almost all models predicted the experimental observations. The poor joint co-variation measured for P1 and P4 were accounted for by all models, except, of course, the angle jerk and acceleration models for which joint coupling was maximal in all cases (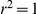). Indeed, for these models, the paths in joint space are straight lines. The energy model tended to over-evaluate the decrease of joint coupling for P1 and P4, because, the optimal movements resulted in only rotating the elbow for P1 and the shoulder for P4, while keeping the other joint frozen. This strategy was produced by some subjects in practice. For instance, they did use a single-joint rotation of the elbow to reach the bar when starting from P1 (8/20 subjects rotated the shoulder less that 10 degrees for P1 and, for every subject, the elbow rotated four times more that the shoulder). The hybrid model performed again well in reproducing the joint coupling across initial postures and subjects. An analysis of Figure 9D showed that the hand jerk and effort models predicted better the joint coupling on average, but since the corresponding finger paths were not realistic, this finding is considered to be irrelevant.

**Figure 10:**
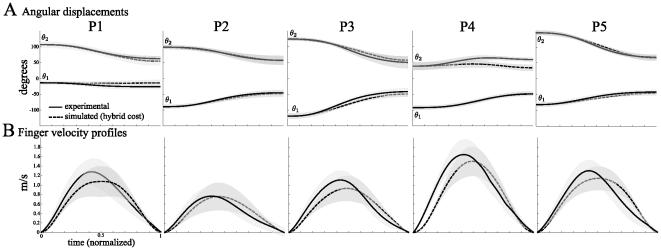
Simulated angular displacements and finger velocity profiles. A. Angular displacements at the shoulder and elbow joints. B. Finger velocity profiles. In both graphs, solid lines correspond to the experimental data, which are recalled from Figure 10 to facilitate comparisons. Dashed lines correspond to the simulated data (averaged across subjects), for the hybrid model, mixing the minimization of the mechanical energy expenditure and the angle acceleration energy. Shaded areas indicate the standard deviation. Time is normalized, but not amplitude.

We also checked that the hybrid model predicted plausible angular displacements and finger velocity profiles. Figure 10A shows that the model (dashed lines) and data traces (solid lines) were globally superimposed, except maybe for posture P4 at the elbow joint. Concerning the finger velocity profiles, Figure 10B shows that they were bell-shaped for all conditions. Note, however, a slight but constant discrepancy between the model predictions and the recorded data. In fact, the deceleration phase was always longer in reality compared to the hybrid model predictions. Nevertheless, even the minimum hand jerk model, which is usually considered as one of the best model for predicting the time-course of the end-effector, would also exhibit the same discrepancy.

Finally, the observed movement variability shows that the behavior of subjects was in fact approximately optimal on a trial-to-trial basis. Figure 7B-I illustrates that there were regions on the bar for which the minimal cost did not vary much (black areas versus white areas). This suggests that, due to the sensorimotor noise and uncertainty, the subjective motor goal could be to keep the movement cost below a certain threshold, as proposed in [46]. In Figure 7, this threshold was set to 10% of the optimal cost in the simulation.

Above all, the modeling analysis showed that the hybrid model, maximizing joint-level smoothness and minimizing mechanical energy expenditure, accounted well for many spatial and temporal features of the observed behaviors, and much better than single cost models (and any other linear cost combination from the inverse optimal control analysis).

## Discussion

In this study we investigated the cost combination hypothesis for the optimal control of arm movements. To this aim we adopted an inverse optimal control methodology to identify the cost function that best replicates the participants' behavior during a task with target redundancy. Inverse optimal control revealed that the observed hand paths were close to the solutions of an optimal control problem relying on a composite cost function mixing mechanical energy expenditure and joint smoothness. This hybrid cost was found to fit well the experimental data, not only much better than any single other cost under comparison, but also better than any other linear combination of the candidate costs.

### On the reaching-to-a-bar paradigm and inverse optimal control

Reaching to objects involving target redundancy is a very common task in everyday life. For instance, grasping a small ball can be achieved through many task-equivalent solutions, depending on how one chooses to put his fingers on it. In such a case, like for the bar, target point discriminability is greatly reduced and, therefore, decision confidence in the brain decreases [47]. Decision making in such a motor planning context [48], [49] can be essentially driven by optimal control [8]. Indeed, resolving the indeterminacy of action selection through optimal control implies that a specific cost function must be selected. Whereas inverse optimal control was considered as a promising tool to characterize automatically the cost function in motor control [21], very little has been done in the context of goal-directed arm movements. Successful applications of inverse methods have been reported in sensorimotor learning [50], [51], human prehension [52], pointing movements [34]. To test the cost combination hypothesis for arm movement planning we decided to use a more generic method [25]. The extrinsic redundancy of the task reduced the risk that several classical cost functions (and thus, several combinations of them) might replicate well the recorded data, which may occur if divergent models could not be sufficiently disambiguated. Indeed, being able to discriminate between different cost functions was precisely a pre-requisite to test whether the CNS combines several cost functions. Figure 1 illustrates that the bar reaching paradigm possesses this property. Inverse optimal control gave us the possibility to drastically enlarge the number of a priori functions that are hypothetically minimized by the CNS, which is usually restricted to few candidate functions in classical studies relying on direct optimal control. In a direct approach, a small number of costs is generally compared and the best one is assumed to be actually optimized by the brain. The weakness is the lack of evidence that another cost, with a different biological meaning, could not perform as well or even better. Although our method did not consider every possible cost function, it improved direct approaches by drastically expanding the search space.

Certain limitations however remain such as the uniqueness of the solution and the problem of local minima, which are hardly avoidable in the context of complex non-linear optimal control. Uniqueness of the solution has been addressed recently in static inverse optimization [52], [53], in the context of additive cost functions and linear constraints. Previous theoretical work on inverse methods was developed in other contexts such as (linear) control theory [54] and reinforcement learning [55], [56]. Here, the present problem was so complex that we tackled it empirically by testing multiple restarts of the algorithm and check a posteriori the effectiveness of the solution compared to basis cost functions. The specific set of eight candidate cost functions has been chosen among a set of costs which could be physiologically interpreted. In this sense, other cost functions such as polynomials could have been included to fit the experimental data but understanding the meaning of such abstract costs would have resulted impossible. Instead we exploited the fact that many costs were already proposed in the literature of arm movement planning. The presence of noise and variability in the observed data is an additional source of difficulty for identifying a unique cost using inverse optimization and only “best fitting” approximations can be found in practice. Here we tested two different metrics in the space of trajectories, based on the Cartesian position of markers (a particularly reliable measure in motion capture systems). Actually, which metric to use to compare human and simulated trajectories remains unresolved [17]. Here, the two metrics we chose allowed to greatly minimize the consequence of noise measurement and inter-trial variability, in contrast to other metrics that may try to fit directly the state vector (including more noisy derived signals, e.g. velocities, torques or accelerations). While these quantities are of course crucial to fully specify a motor plan, attempting to replicate those features and introducing additional uncertainty in the data set may not improve the efficiency of the inverse method. Finally, differences across subjects are rarely addressed in optimal control studies because a single cost, valid for all subjects is generally sought. Inverse optimal control can theoretically reveal if the same costs but weighted differently are actually optimized by different subjects or if the cost ingredients are simply not the same.

### On the identification of the composite cost function

Inverse optimal control results showed that most subjects (15/20) adopted a behavior which essentially corresponded to a strict mixture of two subjective costs (absolute work of torques and angular acceleration energy). More precisely, mixing these two costs was found to fit better the observed hand paths than other linear combinations of the eight candidate costs we considered. Each subject could use a different weighting of those two costs but on average their contribution to the total movement cost was roughly the same (about 40% of the total movement cost). These findings were quite robust as confirmed by the results when using an alternative metric (Text S2, Section 4).

Further evidence for mixing energy and smoothness optimality criteria was provided by the direct optimal control analysis. The bar reaching experiment revealed that several previously proposed costs did not generalize well to the present task. In general, it was relatively easy to discriminate between different models. Clearly, the most discrepant model was the minimum torque model, which assumes that the total amount of (squared) torques needed to drive the movement has to reach a minimum. This model was mainly influenced by the maximum exploitation of gravity to reach the bar. The minimum torque change model, which maximizes smoothness in the dynamic space, also predicted non-biological paths since even the movement direction was poorly predicted in most cases. Similarly, the minimum effort model, optimizing the amount of neural input to control the movement, was unable to predict some basic features of the recorded arm trajectories. Other simulations showed that neither modeling agonist/antagonist muscles as low-pass filters nor separating the control of static (gravitational) and dynamic forces (speed-related) could improve drastically the model predictions for this task (large errors on the movement directions were still clear, see Text S2, Section 1). To remove the problem of gravity integration, we also considered the same task but performed in the horizontal plane (Text S2, Section 2). We tested the behavior of 2 subjects when reaching to an horizontal target bar and the results suggest that those models were still less accurate than the energy, hybrid or geodesic models.

Maximizing smoothness at the level of the hand was also found to be generally irrelevant with respect to the geometry of the paths. The minimum hand jerk model predicted to follow the shortest Euclidean path to reach the bar. It is worth mentioning that this model had been validated originally for horizontal movements performed with a robotic device [11], which could have induced this specific motor strategy [57]. We found differently that the geodesic model, which predicts the shortest paths in joint space using the kinetic energy metric, generalized quite well to the current task. This model is elegant and parameter-free and, therefore, it may be considered to be simpler than the composite cost model that we have identified. One can wonder whether the gain of performance using the hybrid cost is worth its complexity. Whatever the answer, it seems that the cost combination hypothesis would still be supported. Indeed, Biess and collaborators demonstrated recently that “geodesic paths in the Riemannian configuration manifold have been identified as least-effort paths [where effort is defined as the amount of torques that are acting on the arm] as well as the optimal solution of the one-parameter family of MSD [Minimum Squared Derivatives] costs in Riemannian space. Hence, these costs do not only maximize smoothness, but simultaneously minimize movement effort and, thus, encode two performance indices [...]” [58].

It is interesting to note that the geodesic model had been initially validated for unconstrained 3D point-to-point movements [31]. These movements involved redundancy but the specification of the exact target to reach in space combined with the musculoskeletal architecture limiting the joint mobility, significantly reduced the space of admissible behaviors. Consequently, only small differences were observed in many cases between the geodesic model and a model simply predicting straight paths in angle space. The task we presented in this study enlarged the differences between these models (as illustrated in Figure 1). While the geodesic model was quite efficient in predicting path curvature, the minimum angle jerk/acceleration models (predicting straight lines in angle space) captured very precisely the final point on the bar. When reaching to a bar, the actual final postures thus corresponded quite accurately to the final point given by the shortest path in intrinsic space equipped with the Euclidean metric. Other movement features however implied that joint co-variation was not the general rule for motor planning. In particular, for certain starting postures, the only means to replicate the shape of finger paths was to include the minimization of the absolute work of torques into the cost. Interestingly, minimizing this mechanical energy expenditure also resulted in final hand positions that were comparable to the real ones. In agreement with the inverse optimal control results, relevant features of the bar reaching task were better reproduced by a composite cost involving two complementary functions. This complementarity revealed itself quite clear with respect to parameters such as hand path curvature and joint coupling. The matching between the hybrid cost model and the real data was however not perfect, notably with respect to the endpoint location. This discrepancy could be due to the role of vision, which may partly influence the endpoint selection process, but this remains to be investigated. We nevertheless checked the predictions of the models with known endpoints in the Text S2 (Section 3) and showed that the hybrid model accurately predicts the trajectories in the case of point-to-point reachings. Another explanation could be related to the fact that the vector 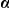 is actually not fixed across conditions but varies depending on the initial posture. This possibility may be suggested by Figure 5B where different cost contributions are obtained for different experimental conditions. The brain may nevertheless prefer to keep constant the respective contributions of complementary costs because of their physical meaning (rather than preserving the way they are combined). This would require adjusting the weighting vector 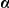 during the planning process to ensure that the resulting movement equally takes into account the different performance criteria, which is a testable hypothesis.

### On the cost combination hypothesis and the optimization of smoothness/energy

It is undeniable that a theory of motor planning assuming that the CNS is able to combine different objectives depending on the task would be very powerful for explaining almost every experimental fact and could be unfalsifiable [10]. Without any prior expectation on the costs that the CNS may combine, it is likely that such a theory would be inappropriate to identify the variables represented by the brain. However, to reduce such a drawback, we propose a more structured view. It is worth noting that the combination of energy and smoothness costs was revealed by a task with reduced external constraints on the target. By extension, we suggest that these costs emerged more clearly because we focused on natural/unconstrained movements. The present results, however, raise a fundamental question: why a combination of energy and smoothness? First, since every movement consumes energy, minimizing its expenditure seems to be an appropriate strategy to keep the musculoskeletal system close to its nominal state. For instance, muscle fatigue alters the execution of actions which might be decisive for species survival. Accordingly, such an optimal behavior may have arisen from natural selection [59], [60]. Second, self-injuring the musculoskeletal system can have dramatic consequences so that pulling a muscle or slipping a joint could have undesirable consequences. Maximizing smoothness therefore contributes also to keep the system close to its operational state. The functional meaning of such costs thus appears related to homeostasis, that is to the process that maintains the internal state of biological systems within bounds [61], [62]. Accordingly, the relevance of such subjective costs had been previously reported for different species and motion, but most of the times these studies focused either on energy or on smoothness. Emphasizing on the mechanical energy, [63] reported evidence that energy was a primary constraint for legged insect locomotion. In a previous study [34], we showed that particular temporal and electromyographic features of vertical pointing movements reflected mechanical energy minimization (i.e. absolute work of torques). Focusing on joint smoothness, [27] showed that a cost function based on the angular acceleration fit well with point-to-point movements in the horizontal plane. Part of the few studies considering composite costs (but using direct optimal control), [64] reported strong evidence for simultaneous multiple performance objectives including the angular acceleration and the mechanical energy expenditure during human locomotion.

The fact that, in this study, energy and smoothness were jointly optimized in roughly similar proportions further supports the relevance of combining subjective costs: minimizing only energy may be detrimental to smoothness and vice-versa. The complementarity of cost functions has been rarely discussed in the motor control literature, even though it constitutes the main motivation for mixing different goals in the same motor plan. For energy and smoothness, the complementarity is evident. However, other costs turn out to be more correlated, in the sense that minimizing the one can imply a decrease of the other. For example, minimizing the amount of motor command (effort cost) may result in a “not so large” torque change cost. Due to nonlinearities, it is nevertheless difficult to establish general rules. In the same vein, the similarity of joint acceleration and jerk costs is the reason why, in this study, we only conclude about the optimization of a quite generic “joint smoothness” term. In general, objective costs are also optimized for the task achievement per se and are thus complementary to subjective costs. Consider for instance the task of drawing a straight line on a sheet on paper. In this case, optimizing the jerk at hand would be the best solution to produce such a path. Energy and joint smoothness costs could however be integrated in the motor plan to determine the remaining degrees of freedom (i.e. joint angles, muscles activities...). Conversely, when trying to jump at a maximal height, it is likely that the weight given to the energy cost is decreased. Joint smoothness should instead remain still present to avoid injuries and fulfill goal achievement. We propose therefore that planning is a dynamic process weighting flexible objective costs (e.g. pointing accuracy, path tracking, via-point etc.) with more deeply anchored subjective costs. This combination of cost would crucially yield the necessary flexibility for the sensorimotor system to achieve a variety of tasks, which agrees with other recent results obtained in the stochastic optimal control context [20].

We still remain ignorant about the detailed neural mechanisms underlying such flexible combinations of cost functions. We may suggest however that subjective cost functions are encoded at a low level of the CNS, while objective cost functions are determined at a higher level. Autonomic motor system that control basic involuntary function through the sympathic system dealing with body's resources might regulate the selection and combination of costs. In other words, we speculate that hypothalamus, reticular formation and spinal cord, which ensure the regulation of internal body states contributing to overall physiological balance, would control the optimization process, however remaining under the influence of descending pathways. Such a hierarchical view of motor planning and control is reminiscent of the theory proposed in [65] where it was suggested that the role of the low-level controller is to compute energy-efficient motor commands that conform to the higher-level variables encoding the constraints of the task itself. Most of the time, external constraints are task-dependent (hand accuracy, speed, center of mass position etc.), while internal constraints may be embodied in the nervous system as subjective constraints resulting from evolutionary, hereditary and learning processes. This proposal needs however to be investigated more deeply. Testing whether the complementary costs we have found are still present when external constraints and explicit rewards strongly shape the motor output could contribute to answer this unresolved question.

## Supporting Information

Text S1 General settings of the optimal control problems and details about their solutions.
(PDF)

Text S2 Materials to verify and support the results described in the main text.
(PDF)
