# Supplementary material for: Evidence for Composite Cost Functions in Arm Movement Planning: An Inverse Optimal Control Approach
Source: PLoS Comput Biol. 2011 Oct 13;7(10):e1002183. doi: 10.1371/journal.pcbi.1002183 (PMC3192804; doi:10.1371/journal.pcbi.1002183)
Supplement: Text S1 — General settings of the optimal control problems and details about their solutions. (PDF) [file pcbi.1002183.s001.pdf]

Text S1

Evidence for composite cost functions in arm movement planning: an inverse optimal control approach

Bastien Berret, Enrico Chiovetto, Francesco Nori and Thierry Pozzo

In this document, we define the general settings used to formulate the optimal control problems (OCPs, Section 1). Section 2 provides insights on how to solve and/or to convert the original models in our framework.

1 General settings and framework

Let us recall that the dynamical system under consideration corresponds to a two-joint and planar arm and is of the form (Σ):

$$\begin{array}{ll} \textit{limb mechanics} & \boldsymbol{\tau} = \mathcal{M}(\boldsymbol{\theta})\ddot{\boldsymbol{\theta}} + \mathcal{C}(\boldsymbol{\theta},\dot{\boldsymbol{\theta}})\dot{\boldsymbol{\theta}} + \mathcal{G}(\boldsymbol{\theta}) + \mathcal{F}\dot{\boldsymbol{\theta}} \\ \textit{actuator dynamics} & \ddot{\boldsymbol{\tau}} = \boldsymbol{\mu} \end{array} \tag{1}$$

More precisely, writing the matrix $\mathcal{M} = (\mathcal{M}_{ij})_{1\leq i,j\leq 2}$, similarly for $\mathcal{F}$ and $\mathcal{C}$ and the vector $\mathcal{G} = (\mathcal{G}_i)_{1\leq i\leq 2}$, we have:

$$\begin{array}{rcl} \mathcal{M}_{11} &=& m_1 l_{c1}^2 + I_1 + m_2 l_{c2}^2 + I_2 + m_2(l_1^2 + 2 l_1 l_{c2}\cos\theta_2), \\ \mathcal{M}_{12} &=& m_2 l_{c2}^2 + I_2 + m_2 l_1 l_{c2}\cos\theta_2, \\ \mathcal{M}_{21} &=& \mathcal{M}_{12}, \\ \mathcal{M}_{22} &=& m_2 l_{c2}^2 + I_2, \\ c &=& m_2 l_1 l_{c2}\sin\theta_2 \\ \mathcal{C}_{11} &=& -2\dot{\theta}_2 c, \\ \mathcal{C}_{12} &=& -\dot{\theta}_2 c, \\ \mathcal{C}_{21} &=& \dot{\theta}_1 c, \\ \mathcal{C}_{22} &=& 0, \\ \mathcal{G}_1 &=& g\{m_1 l_{c1}\cos\theta_1 + m_2(l_{c2}\cos(\theta_1+\theta_2) + l_1\cos\theta_1)\}, \\ \mathcal{G}_2 &=& g m_2 l_{c2}\cos(\theta_1+\theta_2), \\ \mathcal{F}_{ij} &=& \text{constants.} \end{array} \tag{2}$$

The numerical values for the above quantities can be found in Table S1, which comes from Winter's documented tables [1].

notation	description	value and unit
M_s	total mass of the subject	measured, kg
L_s	height of the subject	measured, m
m_1	mass of the upper arm	$\approx M_s \times 0.028$ kg
m_2	mass of the forearm (+hand)	$\approx M_s \times 0.022$ kg
l_1	length of the arm	$\approx 0.186 \times L_s$ m
l_2	length of the forearm	$\approx (0.146 + 0.108) \times L_s$ m
l_{c1}	length from shoulder to center of mass of the arm	$\approx l_1 \times 0.436$ m
l_{c2}	length from shoulder to center of mass of the forearm	$\approx l_2 \times 0.682$ m
I_1	inertia with respect to center of mass of the arm	$\approx m_1 \times (l_1 \times 0.322)^2$ kg.m^2
I_2	inertia of the forearm w.r.t center of mass of the forearm	$\approx m_2 \times (l_2 \times 0.468)^2$ kg.m^2
g	gravity acceleration	≈ 9.81 m.s^{-2}

Table S1: Anthropometric parameters taken from [1] and gravity acceleration.

We also impose the following constraints to keep state and control values within biological bounds:

$$\begin{aligned} -\pi &\le \theta_1 \le \pi\,,\; 0 \le \theta_2 \le \pi \text{ rad}, \\ -10 &\le \dot{\theta}_1, \dot{\theta}_2 \le 10 \text{ rad.s}^{-1}, \\ -100 &\le \tau_1, \tau_2 \le 100 \text{ N.m}, \\ -500 &\le \dot{\tau}_1, \dot{\tau}_2 \le 500 \text{ N.m.s}^{-1}, \\ -5000 &\le \mu_1, \mu_2 \le 5000 \text{ N.m.s}^{-2}. \end{aligned} \tag{3}$$

For a deterministic OCP, Pontryagin's maximum principle (PMP) provides necessary conditions for optimality and the under-determination of the final point is resolved by means of the transversality conditions. When relevant and possible, the PMP was used to verify the validity of the solutions found by the numerical technique we employed ($\mathcal{GPOPS}$) during inverse optimal control. For certain models/costs (trajectory optimization, geodesic...), we also compared those solutions with the ones obtained from the original method, as described by their respective authors. Nevertheless the general and unified framework provided here allowed to compare several existing models and to apply the inverse optimal control algorithm described in the main text.

2 Solutions to the optimal control problems

2.1 Preliminary: necessary conditions for optimality

A general necessary condition for optimality is provided the maximum principle ([2, 3]). We will first neglect the state constraints and just check them a posteriori.

Clarke's version of PMP

Consider a control system $\dot{\mathbf{q}} = \mathbf{f}(\mathbf{q}, \mathbf{u})$ with a cost of the form $C(\mathbf{u}) = \int_0^T g(\mathbf{q}, \mathbf{u})dt$ where f and g are smooth and Lipschitz-continuous functions, respectively. The goal is to connect an equilibrium source point $\mathbf{q}_s$ to an equilibrium point of the target manifold $\mathcal{B} = \{\mathbf{q} \in \mathbb{R}^8$ such that $\mathbf{m}(\mathbf{q}) = \mathbf{0}\}$.

If *the control* $\mathbf{u} \in \mathcal{U}$ *is optimal* ***then*** *there exists a non-trivial absolutely continuous mapping* $(\mathbf{p}(.), \lambda)$: $[0, T] \rightarrow \mathbb{R}^n \times \mathbb{R}^+$ *called adjoint vector, where* λ *is a non-negative constant, such that the trajectory associated to control* $\mathbf{u}$ *verifies the following conditions :*

$$\begin{aligned} \dot{\mathbf{q}} &= \frac{\partial \mathcal{H}}{\partial \mathbf{p}}(\mathbf{q}, \mathbf{p}, \mathbf{u}) \\ \dot{\mathbf{p}} &\in -\partial_{\mathbf{q}} \mathcal{H}(\mathbf{q}, \mathbf{p}, \mathbf{u}) \end{aligned} \tag{4}$$

where $\mathcal{H}(\mathbf{q}, \mathbf{p}, \lambda, \mathbf{u}) =< \mathbf{p}, \mathbf{f}(\mathbf{q}, \mathbf{u}) > +\lambda g(\mathbf{q}, \mathbf{u})$ *is the Hamiltonian of the system, and moreover :*

$$\mathcal{H}(\mathbf{q}, \mathbf{p}, \lambda) = \min_{\mathbf{u}} \mathcal{H}(\mathbf{q}, \mathbf{p}, \lambda, \mathbf{u}) \tag{5}$$

In addition, the transversality condition must hold at the terminal point, i.e., $\mathbf{p}(T) \perp T_{\mathbf{q}(T)}\mathcal{B}$.

In the above statement, $\partial_{\mathbf{q}} \mathcal{H}(\mathbf{q}, \mathbf{p}, \mathbf{u})$ denotes the Clarke's generalized gradient for locally Lipschitz functions, i.e., the convex hull of all possible limits of derivatives of $\mathcal{H}$ at points $\mathbf{q}_n \in \mathbb{R}^8$ with $\mathbf{q}_n \rightarrow \mathbf{q}$ [3]. Note that if all the quantities involved are smooth, the generalized gradient reduces to the classical differentiation.

In our experiment, the target bar was characterized by the following manifold:

$$\mathbf{m}: \ \mathbf{q} = \begin{pmatrix} \theta_1 \\ \theta_2 \\ \dot{\theta}_1 \\ \dot{\theta}_2 \\ \tau_1 \\ \tau_2 \\ \dot{\tau}_1 \\ \dot{\tau}_1 \end{pmatrix} \mapsto \mathbf{m}(\mathbf{q}) = \begin{pmatrix} l_1 \cos\theta_1 + l_2 \cos(\theta_1 + \theta_2) - 0.85L \\ \dot{\theta}_1 \\ \dot{\theta}_2 \\ \tau_1 - \mathcal{G}_1(\theta_1, \theta_2) \\ \tau_2 - \mathcal{G}_2(\theta_1, \theta_2) \\ \dot{\tau}_1 \\ \dot{\tau}_2 \end{pmatrix}. \tag{6}$$

The first row of the mapping $\mathbf{m}$ defines the geometric target bar constraint, while the remaining rows account for the fact that the terminal point is an equilibrium point for the arm dynamics. In particular, the joint torques have to counteract the gravitational torques.

The transversality condition means that the adjoint vector at final time should be a linear combination of the gradient vectors $\frac{\partial \mathbf{m}_1}{\partial \mathbf{q}}_{|\mathbf{q}(T)}, ..., \frac{\partial \mathbf{m}_7}{\partial \mathbf{q}}_{|\mathbf{q}(T)}$ (indeed, since all gradients vectors are orthogonal to the tangent plane at point $\mathbf{q}$). In particular $\det(\mathbf{p}(T), \frac{\partial \mathbf{m}_1}{\partial \mathbf{q}}_{|\mathbf{q}(T)}, ..., \frac{\partial \mathbf{m}_7}{\partial \mathbf{q}}_{|\mathbf{q}(T)}) = 0$ is a necessary and sufficient condition that satisfies the transversality condition.

The maximum principle leads to the problem of finding the values of $\mathbf{p}(t = 0)$ and $\mathbf{p}(t = T)$ that satisfy the conditions $\mathbf{m}(\mathbf{q}) = \mathbf{0}$ and $\mathbf{p}(T) \perp T_{\mathbf{q}(T)}\mathcal{B}$. Note that one has to calculate $\mathbf{u}(\mathbf{q}, \mathbf{p}, \lambda) = \arg\min_{\mathbf{u}} \mathcal{H}(\mathbf{q}, \mathbf{p}, \lambda, \mathbf{u})$ so that, indeed, we obtain a boundary value problem.

2.2 Computation of the optimal solutions for the different models/costs

The numerical inverse optimal control method required formulating different models within a single class of optimal control problems. To this aim, the original models were sometimes slightly modified to fit in a

unified framework. We checked, as described below, that the arm trajectories provided by these reformulated problems were nevertheless consistent and close enough to the original solutions.

2.2.1 Minimum hand jerk, angle jerk, and angle acceleration models

All these problems, involving kinematic costs, are independent on the system dynamics, and were originally formulated as trajectory optimization problems. For instance, the minimum Cartesian hand jerk problem was solved by [4], by applying Euler-Lagrange necessary conditions for optimality.

Consider a minimum jerk problem. Let $\mathbf{v}$ be some variable whose jerk has to be minimized, i.e. we seek for $\mathbf{v} = \arg\min_{\mathbf{v}} \int_0^T ||\frac{d^3\mathbf{v}}{dt^3}||^2 dt$, under the constraint of connecting an equilibrium source point $\mathbf{v}_S$ to an equilibrium terminal point $\mathbf{v}_T$ in time T. Then it is well-known that the optimal path is given by a 5th order polynomial:

$$\mathbf{v}^\star(s) = \mathbf{v}_S + (\mathbf{v}_S - \mathbf{v}_T)(15s^4 - 6s^5 - 10s^3), \tag{7}$$

with $s = \frac{t}{T}$. In a subsequent step, inverse dynamics computations are required if one wants to obtain the corresponding control action or torques.

Moreover, in this study, the terminal state is not a point but a manifold (the bar, denoted by $\mathcal{B}$). We used a straightforward approach to solve this problem, that is to seek for the final point on the bar that yields the minimum cost. To this aim, we solved a non-linear constrained optimization problem. Defining $C(\mathbf{v}_T) = \int_0^T ||\frac{d^3\mathbf{v}^\star}{dt^3}||^2 dt$, the terminal point on the bar is simply given by $\mathbf{v}_T = \arg\min_{\mathbf{v}_T} C(\mathbf{v}_T)$. The constraint is that the terminal point on the bar has to be within the reachable region of the arm. In fact, it is seen that these models lead to the shortest possible path to the manifold, in the space on which the variable $\mathbf{v}$ is lying.

It is worth noting that the above optimal trajectory $\mathbf{v}$ is not necessarily admissible for the dynamical system (Σ). In fact, in the framework provided by the system (Σ), these models could also be treated as classical OCPs. Therefore, we also solved these OCPs using numerical techniques for optimal control (such as $\mathcal{GPOPS}$, [5]). These two methods yielded identical solutions for what concerned the finger paths.

2.2.2 Geodesic model

Following the original formulation of [6], we treated the problem by separating its spatial and temporal aspects. Firstly, geodesics are found by solving a boundary value problem. Here geodesics are meant with respect to the kinetic energy metric of the arm. Secondly, the time course of the finger is derived from a minimum jerk velocity profile. Then, to solve our particular reaching-to-a-bar problem, we discretized the bar every 0.5 cm and solved the problem for all points, similarly to what was done for 3-D arm movements in [6] (in which the problem of free final point occurred as well). We selected the best terminal point, i.e. the one associated to the minimal geodesic length.

As pointed out previously, the optimal solution obtained using this method may not be admissible for the system (Σ). Therefore, we also formulated this problem as an OCP and solved it numerically using $\mathcal{GPOPS}$. This led to the same geometric paths (although not the same time courses).

2.2.3 Smooth OCPs: the minimum torque change example

In this subsection, we deal with smooth OCPs which can be solved by applying the PMP. Here and in the next subsection, a sketch of the procedure for establishing the necessary conditions for optimality using the PMP is given.

Before going further, let us rename the variables as follows:

$$\begin{gathered} x_1 = \theta_1, y_1 = \dot{\theta}_1, x_2 = \theta_2, y_2 = \dot{\theta}_2, z_1 = \tau_1, z_2 = \tau_2, w_1 = \dot{\tau}_1, w_2 = \dot{\tau}_2, \\ u_1 = \mu_1, u_2 = \mu_2. \end{gathered} \tag{8}$$

The control system can thus be rewritten as:

$$\left\{ \begin{array}{l} \dot{x}_1 = y_1 \\ \dot{x}_2 = y_2 \\ \begin{array}{c} \dot{y}_1 \\ \dot{y}_2 \end{array} = \mathcal{M}^{-1} \left(\left(\begin{array}{c} z_1 + c(y_2^2 + 2y_1 y_2) - \mathcal{G}_1 \\ z_2 - c y_1^2 - \mathcal{G}_2 \end{array} \right) - \mathcal{F} \left(\begin{array}{c} y_1 \\ y_2 \end{array} \right) \right) \\ \dot{z}_1 = w_1 \\ \dot{z}_2 = w_2 \\ \dot{w}_1 = u_1 \\ \dot{w}_2 = u_2 \end{array} \right. . \tag{9}$$

For any x_2, $\mathcal{M}$ is always invertible. We set

$$\mathcal{M}^{-1} = \left(\begin{array}{cc} \bar{\mathcal{M}}_{11} & \bar{\mathcal{M}}_{12} \\ \bar{\mathcal{M}}_{21} & \bar{\mathcal{M}}_{22} \end{array} \right). \tag{10}$$

The explicit expression of the elements of $\mathcal{M}^{-1}$ is:

$$\begin{aligned} d \;=\;& m_1 l_{c1}^2 m_2 l_{c2}^2 + (m_1 l_{c1}^2 + m_2 l_1^2) I_2 + I_1 (m_2 l_{c2}^2 + I_2) \\ & + m_2^2 l_1^2 l_{c2}^2 (1 - \cos^2 x_2), \end{aligned} \tag{11}$$

and:

$$\begin{aligned} \bar{\mathcal{M}}_{11} &= \frac{m_2 l_{c2}^2 + I_2}{d}, \\ \bar{\mathcal{M}}_{12} &= \bar{M}_{21}(x_2) = -\frac{m_2 l_{c2}^2 + m_2 l_1 l_{c2} \cos x_2 + I_2}{d}, \\ \bar{\mathcal{M}}_{22} &= \frac{m_1 l_{c1}^2 + I_1 + m_2 l_1^2 + m_2 l_{c2}^2 + 2 m_2 l_1 l_{c2} \cos x_2 + I_2}{d}. \end{aligned} \tag{12}$$

Following the current notations, the integral cost for the minimum torque change is:

$$C_4 = \int_0^T w_1^2 + w_2^2 dt. \tag{13}$$

The Hamiltonian function is:

$$\begin{aligned}\mathcal{H} &= \lambda\{w_1^2+w_2^2\}+p_1y_1+p_2y_2+q_1\dot{y}_1+q_2\dot{y}_2 \\ &\quad +r_1w_1+r_2w_2+s_1u_1+s_2u_2.\end{aligned} \tag{14}$$

As usual in the resolution of OCPs, singular extremals ($\lambda = 0$) and regular extremals have to be considered. Let us recall that a bang extremal is an extremal such that, for almost all time, one of the control component, u_i, can take the two values $u_i^{\max}$ or $u_i^{\min}$ only. Here, it can be shown that singular extremals are necessarily bang (in other words, there is no abnormal extremal). In particular, for an abnormal extremal, the adjoint vector would vanish which would contradict the PMP. Indeed, the following chain of implications would occur: $\mathbf{s} \equiv 0 \Rightarrow \mathbf{r} \equiv 0 \Rightarrow \mathbf{q} \equiv 0 \Rightarrow \mathbf{p} \equiv 0$ (the key point being the invertibility of $\mathcal{M}$). There are thus only two kinds of extremals in this OCP: singular bang extremals, associated with discontinuous controls, and regular extremals. Next, we consider regular extremals. By a standard argument of normalization for the adjoint vector, we can set $\lambda = 1$.

In that case the adjoint equations are:

$$\begin{cases} \dot{p}_1 = -\frac{\partial\mathcal{H}}{\partial x_1} = -q_1\frac{\partial\dot{y}_1}{\partial x_1} - q_2\frac{\partial\dot{y}_2}{\partial x_1} \\ \dot{p}_2 = -\frac{\partial\mathcal{H}}{\partial x_2} = -q_1\frac{\partial\dot{y}_1}{\partial x_2} - q_2\frac{\partial\dot{y}_2}{\partial x_2} \\ \dot{q}_1 = -\frac{\partial\mathcal{H}}{\partial y_1} = -p_1 - q_1\frac{\partial\dot{y}_1}{\partial y_1} - q_2\frac{\partial\dot{y}_2}{\partial y_1} \\ \dot{q}_2 = -\frac{\partial\mathcal{H}}{\partial y_2} = -p_2 - q_1\frac{\partial\dot{y}_1}{\partial y_2} - q_2\frac{\partial\dot{y}_2}{\partial y_2} \\ \dot{r}_1 = -\frac{\partial\mathcal{H}}{\partial z_1} = -q_1\frac{\partial\dot{y}_1}{\partial z_1} - q_2\frac{\partial\dot{y}_2}{\partial z_1} \\ \dot{r}_2 = -\frac{\partial\mathcal{H}}{\partial z_2} = -q_1\frac{\partial\dot{y}_1}{\partial z_2} - q_2\frac{\partial\dot{y}_2}{\partial z_2} \\ \dot{s}_1 = -\frac{\partial\mathcal{H}}{\partial w_1} = -r_1 - 2w_1 \\ \dot{s}_2 = -\frac{\partial\mathcal{H}}{\partial w_2} = -r_2 - 2w_2 \end{cases}. \tag{15}$$

A necessary condition for optimality is the minimization of $\mathcal{H}$ along a trajectory. The optimal control has to satisfy: $(u_1, u_2) = \arg\min_{u_1,u_2} \mathcal{H}(x_1, \ldots, w_2, p_1, \ldots, s_2, u_1, u_2)$. Note that for autonomous systems and fixed time the Hamiltonian remains constant along the optimal trajectory.

When a component of the commutation function is not zero, the control is bang and simply given by:

$$\begin{aligned} u_i &= u_i^{\max} \quad if\, s_i < 0, \\ &= u_i^{\min} \quad if\, s_i > 0. \end{aligned} \tag{16}$$

However, it could happen that $s_i \equiv 0$ on a non-trivial time interval. In such a case, we would get $\dot{s}_i \equiv 0$ and thus, from Equation 15: $w_i \equiv -\frac{1}{2}r_i$.

But $u_i = \dot{w}_i$ from Equation 9. Therefore, we get:

$$\begin{aligned} u_i &= -\tfrac{1}{2}\dot{r}_i \\ &= \tfrac{1}{2}(q_1\tfrac{\partial\dot{y}_1}{\partial z_i} + q_2\tfrac{\partial\dot{y}_2}{\partial z_i}). \end{aligned} \tag{17}$$

Consequently, regular extremals may switch from bang pieces of trajectories to non-bang ones, at times t where $(s_i(t), \dot{s}_i(t)) = (0, 0)$. In general a switching time t results from the condition $s_i(t) = 0$. Here, the control vector (u_1, u_2) might have discontinuities. Notice that, in contrast, the state and adjoint vectors are continuous, just as classical solutions of differential equations and assuming that the boundary values on the state variables are not active (which is checked *a posteriori*).

In practice, we solved this problem with high precision by using the following procedure:

- Initialize the shooting method with a good guess of the optimal solution structure (adjoint vector, commutation times, control sequence etc.). Note that in practice a shooting problem can reveal itself a difficult computational challenge because the radius of convergence may be quite small. The standard approach is to initialize the shooting method by using a guess arising from a numerical optimal control technique.
- Use a Gauss-Newton method to solve the resulting two-point boundary value problem, in which the unknowns are the final adjoint vector, the commutation times, and the terminal position on the bar.

The optimal solution for the starting posture P3 is depicted in Figure S1. It is clear that the necessary conditions are met with a very good precision. Since we only found one extremal meeting these conditions, this extremal was expected to be the global optimal solution. Numerical simulations using $\mathcal{GPOPS}$ confirmed this result and revealed that the numerical method was accurate to find the arm trajectories to the bar.

Similar analytical calculations can be performed for the minimum torque and effort models. However, for costs including the absolute work, i.e. a nonsmooth cost, the scenario can be generally more complex as explained below.

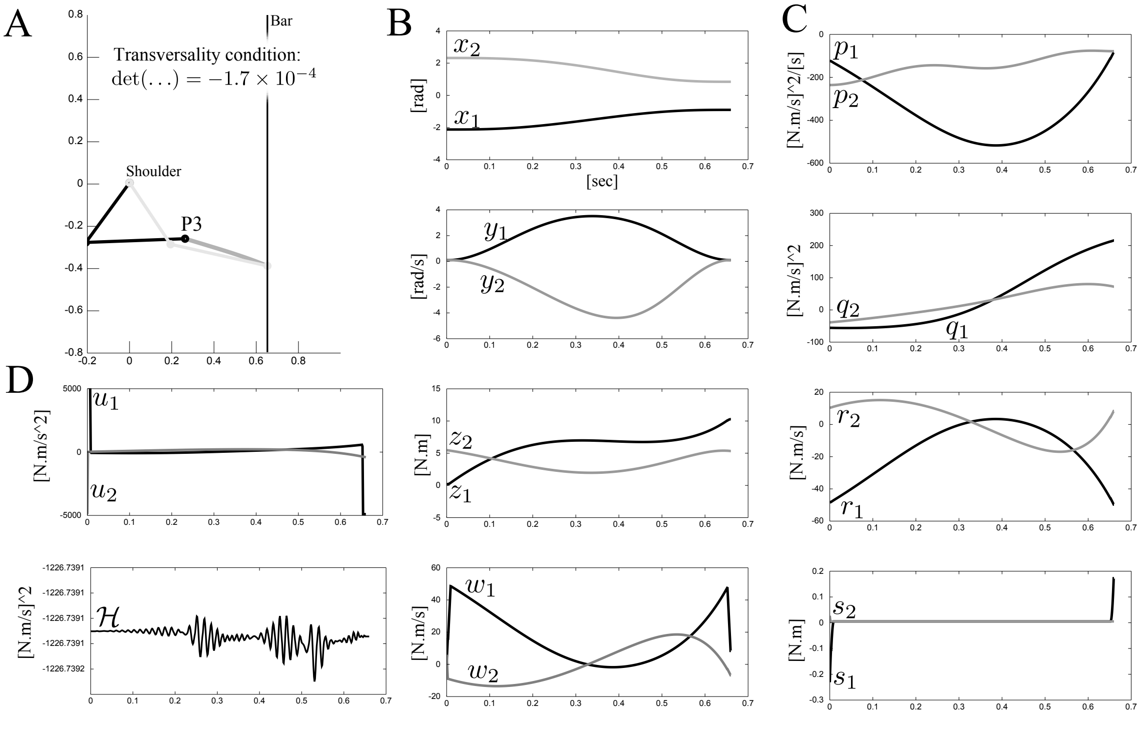

Figure S1: Illustration of the results given by the PMP for the minimum torque change model. A. Stick diagram and finger path. One can check that the terminal adjoint vector is orthogonal to the tangent plane to the manifold at the terminal state. B. State time-series. C. Costate (adjoint) time-series. Note that the commutation functions given by s_1 and s_2 are in fact equal to zero during the main part of the motion. D. Control and Hamiltonian function. One can also check that the Hamiltonian is constant and that the control strategy is of type "bang-regular-bang" (with very short bang pieces of trajectory).

2.2.4 Nonsmooth OCPs: costs including the minimum absolute work.

Considering nonsmooth costs (i.e. not differentiable) substantially complicates the problem of solving an optimal control problem. However, non-differentiality arises naturally when computing the mechanical energy expenditure of a movement. This involves a quantity such as the absolute work of torques (for the energy and hybrid models). A comprehensive mathematical study was provided by [7] for costs involving the absolute work of torques. Such costs imply the use of a nonsmooth version of the PMP [3].

Using the same notations as above, the hybrid cost can be written as:

$$C = \int_0^T |z_1 y_1| + |z_2 y_2| + \alpha(\dot{y}_1^2 + \dot{y}_2^2) dt = C_7 + \alpha C_3. \tag{18}$$

The corresponding Hamiltonian function is:

$$\mathcal{H} = \lambda\{|z_1 y_1| + |z_2 y_2| + \alpha(\dot{y}_1^2 + \dot{y}_2^2)\} + p_1 y_1 + p_2 y_2 + q_1 \dot{y}_1 + q_2 \dot{y}_2 + r_1 w_1 + r_2 w_2 + s_1 u_1 + s_2 u_2. \tag{19}$$

Again, it can be shown that singular extremals are normal. Thus there are only two kinds of extremals in this OCP: singular bang extremals and regular extremals. Next, we consider regular extremals. Let us set $\lambda = 1$, as above.

In that case the adjoint equations of Clarke's extension of the PMP are:

$$\begin{cases} \dot{p}_1 = -\frac{\partial \mathcal{H}}{\partial x_1} = -2\alpha(\dot{y}_1 \frac{\partial \dot{y}_1}{\partial x_1} + \dot{y}_2 \frac{\partial \dot{y}_2}{\partial x_1}) - q_1 \frac{\partial \dot{y}_1}{\partial x_1} - q_2 \frac{\partial \dot{y}_2}{\partial x_1} \\ \dot{p}_2 = -\frac{\partial \mathcal{H}}{\partial x_2} = -2\alpha(\dot{y}_1 \frac{\partial \dot{y}_1}{\partial x_2} + \dot{y}_2 \frac{\partial \dot{y}_2}{\partial x_2}) - q_1 \frac{\partial \dot{y}_1}{\partial x_2} - q_2 \frac{\partial \dot{y}_2}{\partial x_2} \\ \dot{q}_1 \in -\frac{\partial \mathcal{H}}{\partial y_1} = -2\alpha(\dot{y}_1 \frac{\partial \dot{y}_1}{\partial y_1} + \dot{y}_2 \frac{\partial \dot{y}_2}{\partial y_1}) + \mathcal{I}|z_1| - p_1 - q_1 \frac{\partial \dot{y}_1}{\partial y_1} - q_2 \frac{\partial \dot{y}_2}{\partial y_1} \\ \dot{q}_2 \in -\frac{\partial \mathcal{H}}{\partial y_2} = -2\alpha(\dot{y}_1 \frac{\partial \dot{y}_1}{\partial y_2} + \dot{y}_2 \frac{\partial \dot{y}_2}{\partial y_2}) + \mathcal{I}|z_2| - p_2 - q_1 \frac{\partial \dot{y}_1}{\partial y_2} - q_2 \frac{\partial \dot{y}_2}{\partial y_2} \\ \dot{r}_1 \in -\frac{\partial \mathcal{H}}{\partial z_1} = -2\alpha(\dot{y}_1 \frac{\partial \dot{y}_1}{\partial z_1} + \dot{y}_2 \frac{\partial \dot{y}_2}{\partial z_1}) + \mathcal{I}|y_1| - q_1 \frac{\partial \dot{y}_1}{\partial z_1} - q_2 \frac{\partial \dot{y}_2}{\partial z_1} \\ \dot{r}_2 \in -\frac{\partial \mathcal{H}}{\partial z_2} = -2\alpha(\dot{y}_1 \frac{\partial \dot{y}_1}{\partial z_2} + \dot{y}_2 \frac{\partial \dot{y}_2}{\partial z_2}) + \mathcal{I}|y_2| - q_1 \frac{\partial \dot{y}_1}{\partial z_2} - q_2 \frac{\partial \dot{y}_2}{\partial z_2} \\ \dot{s}_1 = -\frac{\partial \mathcal{H}}{\partial w_1} = -r_1 \\ \dot{s}_2 = -\frac{\partial \mathcal{H}}{\partial w_2} = -r_2 \end{cases}, \tag{20}$$

with $\mathcal{I} = [-1, 1]$, coming from Clarke's generalized gradient.

Here, the nonsmooth maximum principle leads to differential inclusions for the adjoint vector at points where $\mathcal{H}$ is not continuously differentiable. These points are those for which $y_i z_i$ vanishes (i.e., when the power of torques becomes zero). The optimal scenario can be quite complex because there may exist pieces of trajectory such that $y_i \equiv 0$ and/or $z_i \equiv 0$, for which only inclusions are given by the maximum principle. In such a case, higher order derivatives of y_i and z_i should be considered to get the control expression.

However, based on the exact solutions derived for the torque-control case in [8] and on numerical simulations, it appears that the optimal strategy is generally similar to what happens for the minimum torque change model presented above: constraints on the control are active only at the beginning and at the end of the movement, and last for a very brief period of time if $u_i^{\max}$ and $u_i^{\min}$ are large enough. Thus, most of the time, the optimal trajectory is a regular extremal and, in particular, we have $s_i \equiv 0$. Thus, u_i can be determined using the fact that $r_i \equiv 0$ and that, in turn, $\dot{r}_i = 0 \in -2\alpha(\dot{y}_1 \frac{\partial \dot{y}_1}{\partial z_i} + \dot{y}_2 \frac{\partial \dot{y}_2}{\partial z_i}) + \mathcal{I}|y_i| - q_1 \frac{\partial \dot{y}_1}{\partial z_i} - q_2 \frac{\partial \dot{y}_2}{\partial z_i}$, along such a

piece of trajectory. This inclusion is an equality if $z_i \neq 0$. The control u_i can be determined by differentiating the latter expression. Otherwise, $z_i \equiv 0$ and, thus, a necessary condition for optimality is that zero belongs to a certain time-varying interval. This was referred to as an “inactivation” in [8]. In that case, we have necessarily $w_i \equiv 0$ and, in turn, $u_i \equiv 0$.

Remark: When the optimal control is discontinuous, one would rather avoid the use of collocation methods since they rely on global polynomial interpolation. An alternative method is to use a direct transcription of the OCP (with a trapezoidal Euler's discretization scheme for instance). In that case, the control is approximated by a piecewise constant function. Nevertheless, we noted that collocation methods yielded quite precise finger trajectories by comparing with the exact solutions derived from the PMP. In fact, the error only increased significantly for higher order variables such as for the torque change or control vectors but we focused our analysis on the hand paths and velocities. However, it was still possible to guess the optimal scenario, allowing in turn the use of the shooting method described above. Finally, in order to deal with nonsmooth costs using numerical techniques, the absolute value function was replaced by the smooth function: $z \mapsto \tanh(10z)z$. This is because NLP methods are generally gradient-based.

References

[1] Winter D (1990) Biomechanics and Motor Control of Human Movement. New York: John Wiley & Sons.

[2] Pontryagin LS, Boltyanskii VG, Gamkrelidze RV, Mishchenko EF (1964) The Mathematical Theory of Optimal Processes. Pergamon Press, 360 pp.

[3] Clarke F (1983) Optimisation and nonsmooth analysis. John Wiley.

[4] Flash T, Hogan N (1985) The coordination of arm movements: an experimentally confirmed mathematical model. J Neurosci 5:1688–1703.

[5] Rao AV, Benson DA, Darby CL, Patterson MA, Francolin C, et al. (2010) Algorithm 902: Gpops, a matlab software for solving multiple-phase optimal control problems using the gauss pseudospectral method. ACM Trans Math Softw 37:1–39.

[6] Biess A, Liebermann DG, Flash T (2007) A computational model for redundant human three-dimensional pointing movements: integration of independent spatial and temporal motor plans simplifies movement dynamics. J Neurosci 27:13045–13064. doi:10.1523/JNEUROSCI.4334-06.2007. URL http://dx.doi.org/10.1523/JNEUROSCI.4334-06.2007.

[7] Gauthier JP, Berret B, Jean F (2010) A biomechanical inactivation principle. Proceedings of the Steklov Institute of Mathematics 268:93–116.

[8] Berret B, Darlot C, Jean F, Pozzo T, Papaxanthis C, et al. (2008) The inactivation principle: mathematical solutions minimizing the absolute work and biological implications for the planning of arm movements. PLoS Comput Biol 4:e1000194. doi:10.1371/journal.pcbi.1000194. URL http://dx.doi.org/10.1371/journal.pcbi.1000194.
