# Supplementary material for: Evidence for Composite Cost Functions in Arm Movement Planning: An Inverse Optimal Control Approach
Source: PLoS Comput Biol. 2011 Oct 13;7(10):e1002183. doi: 10.1371/journal.pcbi.1002183 (PMC3192804; doi:10.1371/journal.pcbi.1002183)
Supplement: Text S2 — Materials to verify and support the results described in the main text. (PDF) [file pcbi.1002183.s002.pdf]

Text S2

Evidence for composite cost functions in arm movement planning: an inverse optimal control approach

Bastien Berret, Enrico Chiovetto, Francesco Nori and Thierry Pozzo

In this document, we provide some materials to verify the robustness of the results described in the main text. In particular, we discuss the effects of modeling differently the actuator dynamics (Section 1), we report the predictions of the models in the horizontal plane (in the absence of gravity, Section 2) and we depict the predictions of the models when the endpoint is given (Section 3). Finally, we consider possible variants for the inverse optimal control method (Section 4). This document essentially illustrates that the conclusions of the main text do not critically depend on the choices we had to make.

1 Sensitivity of the effort model with respect to the actuator dynamics

The effort model (cost C_8, see the main text) can be sensitive to the model of the actuator dynamics. Intuitively, the effort cost aims at representing the amount of neural command that eventually generates the movement of the limb. In the main text, we used a simple model assuming a mechanical actuator, in which the torque acceleration is directly controlled, namely:

$$1st\,actuator\,dynamics \qquad \ddot{\boldsymbol{\tau}} = \boldsymbol{\mu} \ . \tag{1}$$

This is a simplification because in reality the relationship between the neural inputs and the torques produced at a joint is a rather complex dynamical process. A classical yet simple model of torque generation is given by the following 2nd order linear low-pass filter:

$$2nd\,actuator\,dynamics \qquad \begin{array}{l} \dot{\boldsymbol{\tau}} = (\boldsymbol{\epsilon} - \boldsymbol{\tau})/\sigma_1 \\ \dot{\boldsymbol{\epsilon}} = (\boldsymbol{\mu} - \boldsymbol{\epsilon})/\sigma_2 \end{array} \ . \tag{2}$$

In the above equation, $\boldsymbol{\epsilon}$ can be thought as a quantity similar to the electromyographic activity and the constants σ_i denotes time constants characterizing the muscle contraction process. Note that the dimensionality of the optimal control problem (OCP) is not increased in this case: we simply switch from a direct control of the joint torque accelerations to a control of torques through a delayed dynamical process. A fundamental difference between Equation 1 and Equation 2 is the presence of tuning parameters in the latter one. Standard values for the time constants are about 40 ms for the fast arm movements we consider. Another crucial difference is related to the influence of gravity. The first model suggests that keeping a constant arm posture ($\boldsymbol{\tau}_{\text{grav}} \equiv$ constant) is costless (i.e. it can be achieved with $\boldsymbol{\mu} \equiv 0$, that is no effort). In contrast,

the second model states that maintaining a fixed posture has a cost related to the magnitude of $\boldsymbol{\tau}_{\text{grav}}$ (i.e. $\boldsymbol{\mu} \equiv$ constant). This difference is not innocent and suggests that the optimal solutions for vertical movements may differ a lot, as we will illustrate below. A common strategy to circumvent this potential issue is to apply the so-called *separation principle.* This principle allows to solve the optimal control problem without static forces (here gravity) and then to compensate for them during movement execution [1]. However, this is somewhat problematic for inverse optimal control because some models are not compatible with this specific assumption. Working in the horizontal plane obviously removes this type of issue (see Section 2).

Both previous models of the actuator dynamics neglect the agonist/antagonist nature of muscular system. It is possible to model this, up to a significant increase of dimensionality and number of tuning parameters. This dimensionality augmentation is obviously detrimental to the inverse optimal control (both in terms of speed and accuracy of the inner loop, solving direct OCPs). In the simplest case, let us consider a pair of agonist/antagonist muscles at each joint. We follow [1] and, therefore, we consider the following actuator dynamics:

$$\textit{3rd actuator dynamics} \qquad \begin{array}{l} \tau_i = \rho(T_{i1} - T_{i2}) + \mathcal{G}_i \ \ , i \in \{1,2\} \\ T_{ij} = \log(1+\exp(h\alpha_{ij}))/h \ \ , \, i,j \in \{1,2\} \\ \dot{\alpha}_{ij} = (\epsilon_{ij} - \alpha_{ij})/\sigma_{ij} \ \ , \, i,j \in \{1,2\} \\ \dot{\epsilon}_{ij} = (\mu_{ij} - \epsilon_{ij})/\delta_{ij} \ \ , \, i,j \in \{1,2\} \end{array} . \tag{3}$$

The state vector is now 12-D and defined as: $\mathbf{x} = (\theta_1, \theta_2, \dot{\theta}_1, \dot{\theta}_2, \alpha_{11}, \alpha_{12}, \alpha_{21}, \alpha_{22}, \epsilon_{11}, \epsilon_{12}, \epsilon_{21}, \epsilon_{22})^\top$ and the control vector becomes 4-D: $\mathbf{u} = (\mu_{11}, \mu_{12}, \mu_{21}, \mu_{22})^\top$. The first line of Equation 3 states that the net shoulder and elbow torques (τ_1 and τ_2) result from the difference between the tensions exerted by antagonist muscles (T_{i1} and T_{i2}, $i = 1..2$). The factor ρ is used to convert muscle tensions to torques and is considered constant for simplicity. The separation principle is also applied, meaning that dynamic and static torques are separated. Essentially, this implies that gravity cannot not influence movement planning, because the gravitational torques are just added a posteriori to compensate the presence of gravity during execution. The remaining equations describe the dynamics of the 4 muscles (2 at each joint). The subscript ij denotes the muscle j at joint i. The function $z \mapsto \log(1+\exp(hz))/h$ is a smooth function (approximately) returning zero for negative inputs and identity for positive inputs (h is a constant). This emulates the fact that muscles can only pull. The parameters σ_{ij} and δ_{ij} are constants determining the response time of each muscle. For our simulations, parameters are the same as in [1].

Figure S2 illustrates the sensitivity of the effort model to the different models of the actuator dynamics. In general, the separation principle is required to get realistic trajectories, similarly to what was also observed for the minimum torque model in the main text. However, using the separation principle was impossible in our case because this assumption is incompatible with other models that really exploit gravity during trajectory planning (e.g. the energy model). Therefore, it turns out that assuming actuator dynamics given by Equation 1 is a reasonable choice to approximate the effort model and to permit us using the inverse optimal control method. Figure S2 shows that even the best variant of the effort model performs worse than the hybrid model proposed in the main text.

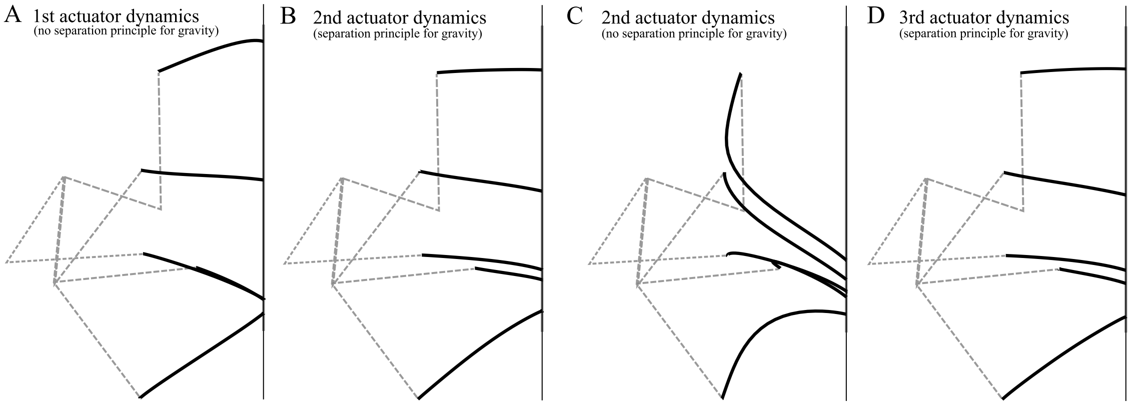

Figure S2: Influence of the actuator dynamics on the effort cost. A. 1st actuator dynamics (Eq. 1), used in the main text. B. 2nd actuator dynamics based on 2nd order low-pass filters (Eq. 2). The separation principle is applied to remove the effects of gravity. C. 2nd actuator dynamics when the separation principle is not applied. A strong influence of gravity is observable in this case. D. 3rd actuator dynamics (Eq. 3) modeling a pair of antagonist muscles at each joint, using the parameters defined in [1] and the separation principle.

2 Predictions of the models in the horizontal plane

The previous section has illustrated that gravity may play an important role for some models. Moreover, given that we do not model accurately the musculoskeletal system of the arm, it is useful to present the predictions of the different models in the horizontal plane. A precise investigation of the role of gravity in arm movement planning is out of the scope of the current study (but see [2, 3] for more detailed discussions).

Figure S3 gives the predictions of all models in the horizontal plane. It is interesting to note that the energy and hybrid models are quite sensitive to the presence of gravity, notably with respect to the curvature of hand paths.

To qualitatively evaluate the behavior of subjects in the horizontal plane, a control experiment involving two subjects was performed. The main protocol was thus ported to the horizontal plane. Subjects were supporting their own weight, that is, no manipulandum or any other device was used. The results are reported in Figure S4. They revealed that subjects tended to produce straighter hand paths than in the vertical plane (although some curvature was clearly visible from P1 and P4). Also, the endpoints seemed to be better discriminated compared to what happened in the vertical plane. Both subjects exhibited similar behaviors, yet with some differences possibly due, at least partly, to differences in anthropomorphic parameters and movement duration. A visual comparison suggests that the geodesic, energy and hybrid models are quite congruent with the experimental data. Other models seem to be discrepant with the data, such as the torque change or angle acceleration/jerk models. It is noted that the torque model provides much more realistic predictions in the absence of gravity (compare to Figure 7 of the main text), but still fails to predict the correct movement direction from postures P2 and P3 (rightward while it is leftward for all subjects). Finally, the hand jerk seems to be more plausible in the horizontal plane than in the vertical plane even though it is clear that the shortest path was not the solution adopted by subjects in most cases. A complete quantitative analysis, involving more subjects and similar to what we did in the main text, would be necessary to reach conclusions

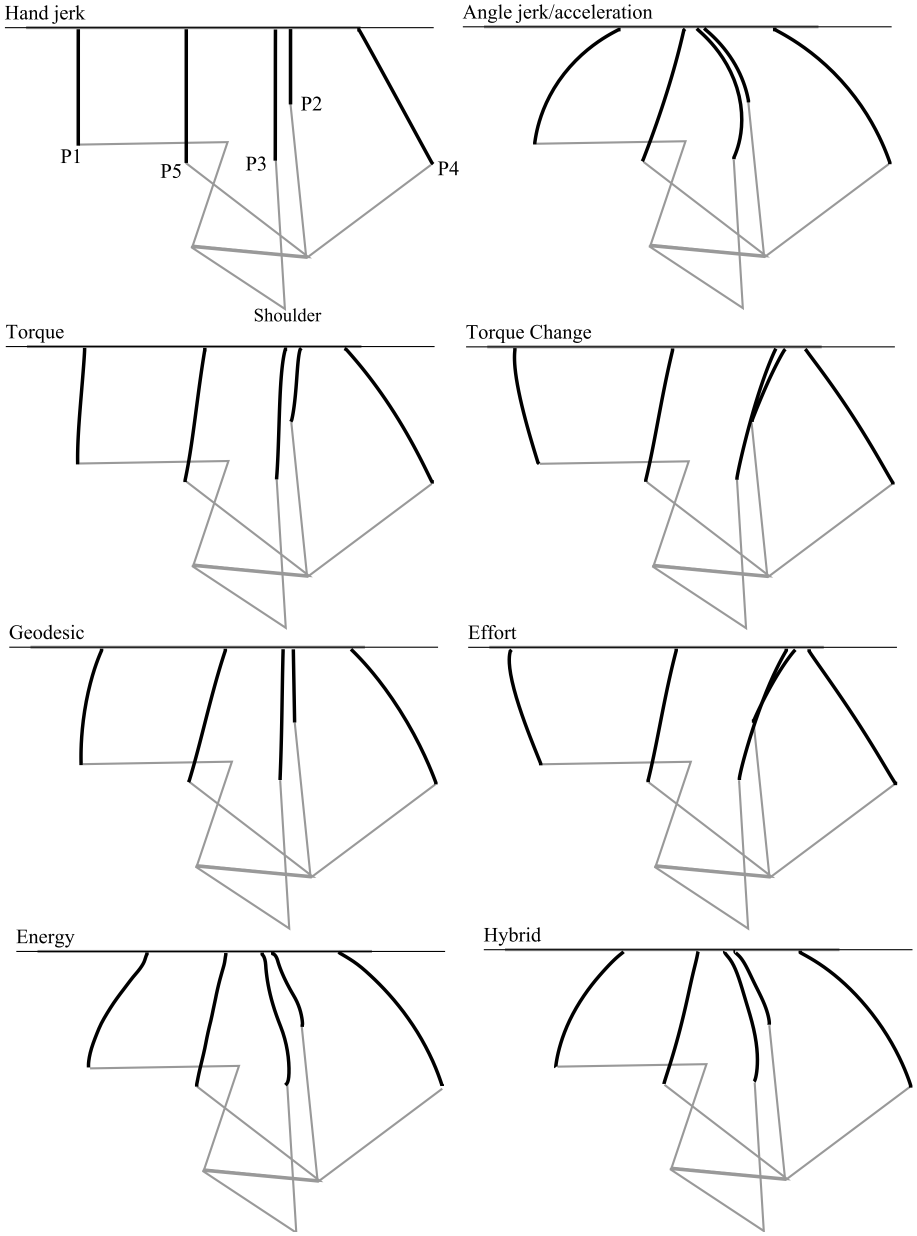

Figure S3: Predictions of the models in the horizontal plane when reaching to a horizontal bar. The hand jerk, angle jerk/acceleration and geodesic models do not depend on gravity and just yield the same predictions as in the vertical plane. The torque, torque change, effort, energy and hybrid models are theoretically gravity dependent, although differences can turn out to be very small (e.g. for the torque change model).

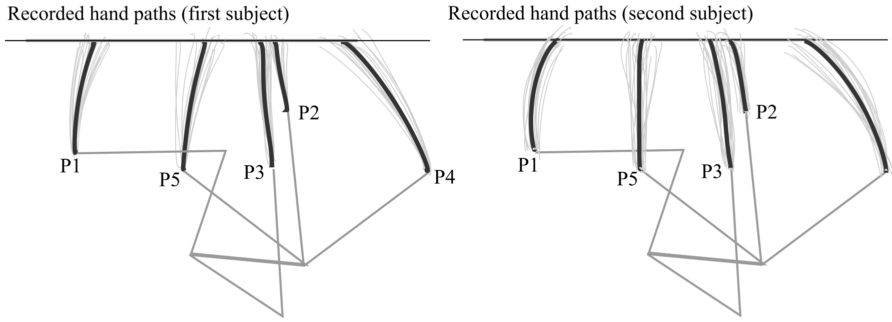

Figure S4: Typical raw data for two subjects when reaching to a bar in the horizontal plane. Five starting postures were tested and 20 trials per starting conditions were recorded.

with a higher level of confidence. These preliminary results nevertheless suggest that the models we found relevant in the vertical plane (i.e. hybrid or geodesic) may generalize quite well to the horizontal plane.

3 Models given the endpoints

In the reaching-to-a-bar paradigm, the endpoint is freely chosen by the subject. Even though we assumed that the endpoint was automatically derived from optimal control, a strategy in which (1) an endpoint is visually selected on the bar and (2) an optimal movement is planned toward that target might be possible. Whatever the strategy used by subjects to select the endpoint, we can nevertheless compare the models when the final point is given. To this aim, we used the data of the typical subject to define the endpoint associated with each starting posture. We then computed the optimal trajectories corresponding to these point-to-point reachings for the different models under consideration.

Figure S5 shows the performance of the various models when the endpoint is known. The hybrid model still performs better than other models and reproduces well all movement conditions ($\triangle_{\text{paths}} = 82\,\text{cm}^2$). The geodesic model also replicates quite well the hand paths ($\triangle_{\text{paths}} = 106\,\text{cm}^2$). This brief analysis demonstrates that, if the two processes that we refer to as "target selection" and "trajectory planning" are separated, the hybrid model matches the experimental data even more accurately. This figure also confirms that using a free endpoint increases the differences between models' predictions. For example here, the hand jerk, geodesic and effort models predict similar hand paths whereas they were clearly distinguishable in the manifold reaching case.

4 Considerations on the inverse optimal control method

4.1 Choice of the metric for the inverse optimal control method

The choice of a "metric" in the space of trajectories still remains under debate. This is nevertheless a crucial choice to compare simulated trajectories with experimental ones and to measure the "degree of similarity"

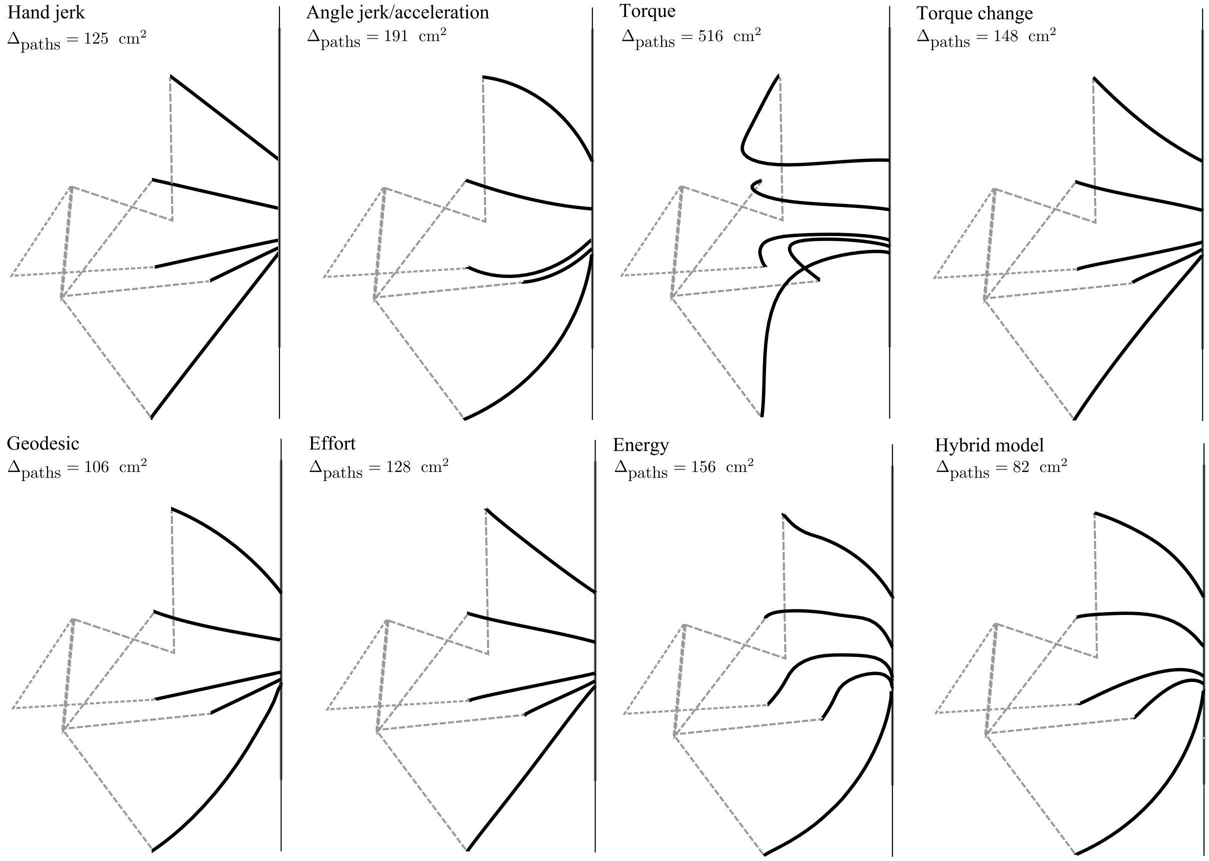

Figure S5: Predictions of the models given the endpoints. Data for the typical subject are illustrated. The $\triangle_{\text{paths}}$ parameter is reported and quantifies the global discrepancy between the simulated trajectories and the experimental one (average across starting postures). The hybrid model is the model obtained after the inverse optimal control procedure for the typical subject.

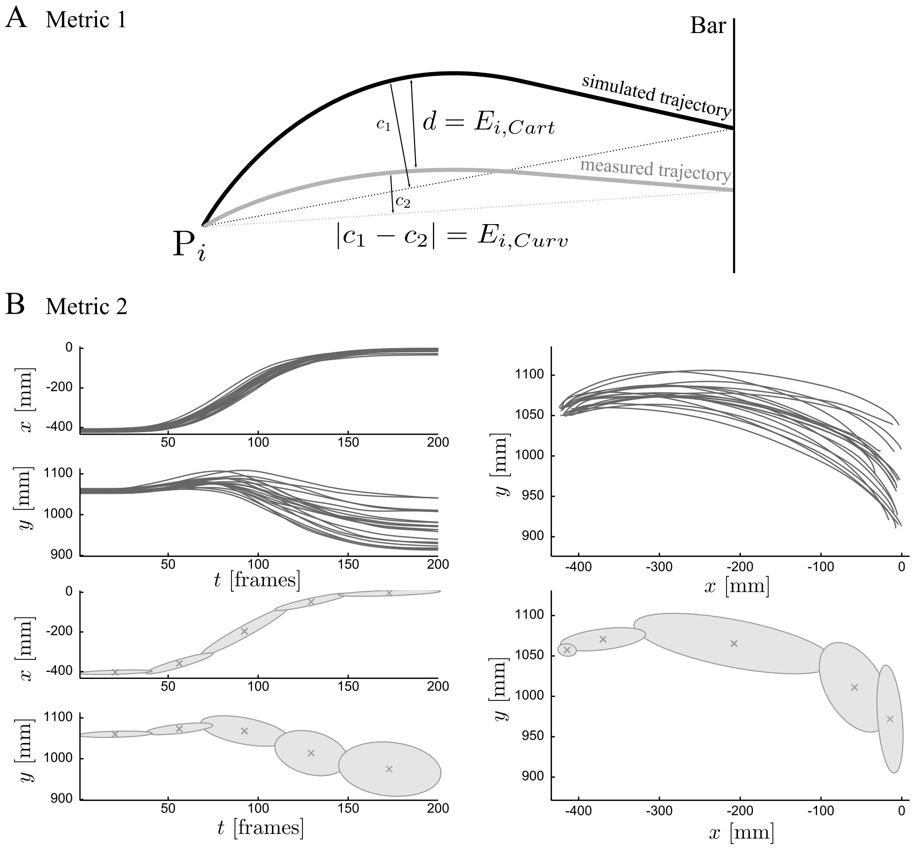

Figure S6: The two metrics under consideration. A. Metric 1, based on Cartesian and curvature errors of the hand paths. This figure assumes that distance d is reported at time $t = \arg\max_t d(t)$ and that c_1 and c_2 are the distances to the straight lines at time $t = \arg\max_t c(t)$. B. Metric 2, based on a statistical model of the human trajectories. The first row shows the endpoint trajectories for all the movements starting from posture P5 of the typical subject. The second row shows the result of fitting a Gaussian Mixture Model to the data (both time t and (x, y) coordinates, with $K = 5$ Gaussians). From this model, a metric is defined via negative log-likelihood computations.

between them. This is especially important for inverse optimal control. In a recent review of models for the generation of multi-joint movements, S. Gielen wrote [4]: "One such problem (not the least important) is that it is not quite clear what metric to use to compare movements along different paths and with different velocity profiles". This metric function can be viewed as a fitting/error function to be minimized through inverse optimal control rather than a metric in a strict mathematical sense. In the main text, we used a metric based on the Cartesian and curvature errors of the hand paths. Alternatively, one can build a statistical model of the experimental data and then compute the likelihood of the simulated trajectories gieven that model, whose results are reported here. The two metrics are described hereafter and illustrated in Figure S6.

4.1.1 Metric 1: Cartesian and curvature errors

In the present study, the first metric function, denoted by Φ_1, was defined as follows:

$$\Phi_1(\mathbf{q}^{\star}_{\boldsymbol{\alpha}}(t), \mathbf{q}^{meas}(t)) = E_{Cart} + E_{Curv}, \tag{4}$$

where:

$$\begin{array}{rcl} E_{Cart} & = & \max\limits_t d(t), \\ \text{where} & & d(t) = \mathrm{d}(\{x^{\star}_{\boldsymbol{\alpha}}(t), y^{\star}_{\boldsymbol{\alpha}}(t)\}; \{x^{meas}, y^{meas}\}) \\ E_{Curv} & = & \max\limits_t c(t), \\ \text{where} & & c(t) = |\mathrm{d}^{\mathrm{s}}(\{x^{\star}_{\boldsymbol{\alpha}}(t), y^{\star}_{\boldsymbol{\alpha}}(t)\}; \{s_{x^{\star}_{\boldsymbol{\alpha}}}, s_{y^{\star}_{\boldsymbol{\alpha}}}\}) - \mathrm{d}^{\mathrm{s}}(\{x^{meas}(t), y^{meas}(t)\}; \{s_{x^{meas}}, s_{y^{meas}}\})|. \end{array} \tag{5}$$

In Equation 5, $(x^{\star}_{\boldsymbol{\alpha}}(t), y^{\star}_{\boldsymbol{\alpha}}(t))$ and $(x^{meas}(t), y^{meas}(t))$ denote the Cartesian trajectories of the finger corresponding to state-space trajectories $\mathbf{q}^{\star}_{\boldsymbol{\alpha}}(t)$ and $\mathbf{q}^{meas}(t)$, respectively. When t is not written explicitly then the path rather than the trajectory is meant. The symbol s stands for the straight path connecting the initial finger position to the final finger position of the corresponding finger path. The operator $\mathrm{d}(\cdot;\cdot)$ returns the orthogonal distance from a point of the trajectory to the path of the measured trajectory defined in the second argument. The operator $\mathrm{d}^s(\cdot;\cdot)$ returns the signed orthogonal distance from a point of the trajectory to the straight path connecting its two extremities (2nd argument), similarly to the definition of sIPC parameter (see the main text, but basically, a plus or minus sign is attributed based on the local concavity and convexity of the path). In this way, if the experimental path is concave while the simulated one is convex, then a larger error will be measured than if both paths are concave (to similar extent of curvatures). The reason for including the curvature as a fitting constraint is the fact that curvature is a quite characteristic feature of human movements and that considering E_{Cart} alone could advantage unrealistic paths oscillating around the measured one. Therefore, both the endpoint position and the shape of the paths were taken into account in the metric Φ_1 (and they had comparable weights, both are simply expressed in meters). The first metric is schematized in Figure S6A.

Note that, in the real simulations, the five starting postures were included in the fitting function, thus forcing the inverse optimal control method to return a single parameter $\boldsymbol{\alpha}$ for all the experimental conditions. The complete metric Φ was thus $\Phi = \frac{1}{5}\sum_{i=1}^{5}\Phi_{i,1} = \frac{1}{5}\sum_{i=1}^{5}E_{i,Cart} + E_{i,Curv}$, the subscript i denoting the starting posture (P1 to P5).

The problem with metric Φ_1 is that it may appear ad-hoc and based on some human intuition. We describe in the next subsection an alternative method to define a relevant metric in the space of trajectories.

4.1.2 Metric 2: Likelihood estimations

Studies on Programming by Demonstration (PbD), in the field of robotics, make extensive use of statistical models to describe human trajectories [5, 6]. Such methods also provide natural metrics in the space of trajectories, via likelihood calculations. Here we use a Gaussian Mixture Model (GMM) to obtain a general description of the experimental trajectories encapsulating all their essential features.

Let us represent a set of experimental trajectories in Cartesian space by the variables $\xi_j = \{t_j, x_j, y_j\}$, $j = 1..N$. This dataset contains N trials for a given condition and each observed trajectory ξ_j is composed of a temporal component t_j (T time steps) and a spatial one (x_j, y_j). More precisely, we represent a single trajectory by $(t, x(t), y(t))$ where $(x(t), y(t))$ are the coordinates of the end-effector in the Cartesian space at time t. This representation includes both time and space in the model. Our aim is then to model the experimental data with a mixture of K Gaussians of dimensionality D.

Given this GMM we have:

$$\begin{aligned}
p(k) &= \pi_k && (\textit{prior}) \\
p(\xi_j|k) &= \mathcal{N}(\xi_j; \mu_k, \Sigma_k) && (\textit{conditional Gaussian pdf}) \\
&= \frac{1}{\sqrt{(2\pi)^D |\Sigma_k|}} \exp(-\tfrac{1}{2}[\xi_j - \mu_k]^\top \Sigma_k^{-1} [\xi_j - \mu_k]) && \\
p(\xi_j) &= \sum_{k=1}^{K} p(k) p(\xi_j|k) && (\textit{total pdf})
\end{aligned}$$

The GMM is thus fully characterized by a set $\lambda = \{\pi_k, \mu_k, \Sigma_k\}_{k=1}^K$. The parameters of λ were estimated via the Expectation-Maximization algorithm, a local search method that guarantees a monotonic increase of the likelihood of the training data [5]. Like most local optimization methods, this algorithm is sensitive to the initial guess, and therefore, a suitable initial guess is desirable. Following [5], a k-means clustering technique was first applied to the data and then, the Gaussian parameters were then derived from the clusters found by the k-means method.

In the present work, we had $N = 20$, $T = 200$, $D = 3$, and we chose $K = 5$ after visual inspection. A graphical illustration of the GMM model obtained from this procedure is given in Figure S6B. We used the open-source Matlab toolbox developed by S. Calinon [5, 6]. The negative log-likelihood of a simulated trajectory given the latter model was eventually computed in order to measure the degree of similarity between simulated and recorded trajectories. To give equal importance to every point, the sampling of a trajectory was performed with respect to arc length and the time was then retrieved from it.

More precisely, we defined the second metric as follows:

$$\Phi_2(\mathbf{q}^\star_{\boldsymbol{\alpha}}(t), \lambda) = -\log \mathcal{L}(x^\star_{\boldsymbol{\alpha}}(t), y^\star_{\boldsymbol{\alpha}}(t); \lambda), \tag{6}$$

where $\mathcal{L}$ is the likelihood of the optimal control trajectory $\{x^\star_{\boldsymbol{\alpha}}(t), y^\star_{\boldsymbol{\alpha}}(t)\}$ given the model λ.

As for metric 1, the complete metric Φ used in the simulations was actually summed over all the initial postures, that is: $\Phi = \frac{1}{5}\sum_{i=1}^{5} \Phi_{i,2} = -\frac{1}{5}\sum_{i=1}^{5} \log \mathcal{L}(x^\star_{\boldsymbol{\alpha}}(t), y^\star_{\boldsymbol{\alpha}}(t); \lambda_i)$.

The results of inverse optimal control using metric 1 were reported in the main text. The results with metric 2 are reported in Figure S7. Globally, the results agree with the ones obtained in the main text, and mainly two costs are pointed out by the method: angle acceleration and energy.

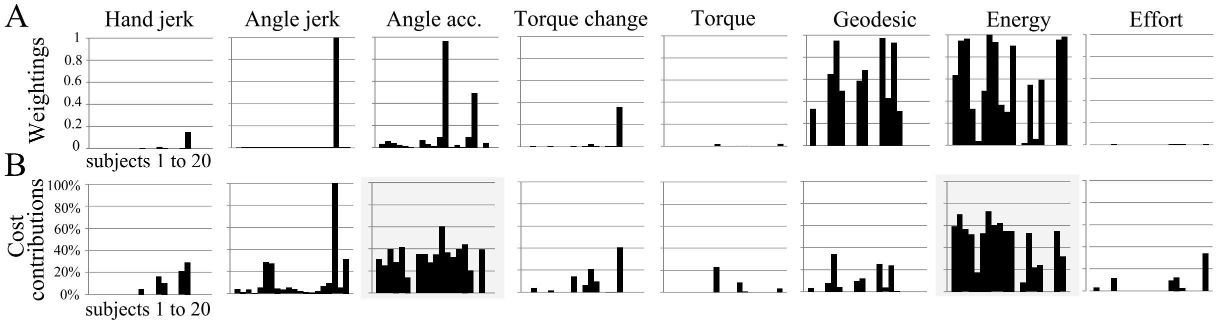

Figure S7: Inverse optimal control results for the 20 subjects using metric 2. A. Weighting coefficients, i.e. elements of the vector $\boldsymbol{\alpha}$ (normalized such that the sum equals 1). Each bar corresponds to one subject. B. Contribution of each cost ingredient to the total cost, for each subject.

4.2 Choice of the re-scaling vector for the inverse optimal control method

The differences in the units of the measure for the combined costs require the use of a re-scaling vector (denoted by $\mathbf{r}$ in the main text). This re-scaling vector is important to properly adjust the size-step along each dimension of the search space. Several strategies may be used to get a suitable re-scaling vector. In the main text, we evaluated the magnitude of the re-scaling vector from multiple simulations of point-to-point movements using single cost functions and by measuring the corresponding optimal movement cost. Another method suggested by a referee could be to determine $\mathbf{r}$ in the spirit of sensitivity analysis. More precisely, one can compute numerically the gradient of the optimal cost $C(\boldsymbol{\alpha})$ (around some point $\boldsymbol{\alpha}$), by evaluating partial derivatives:

$$\frac{\partial C}{\partial \alpha_i} \approx \frac{C(\ldots,\alpha_i,\ldots) - C(\ldots,\alpha_i+\varepsilon,\ldots)}{\varepsilon}, \quad \text{for}\, i \in [1,\ldots,8]. \tag{7}$$

Evaluating this gradient is not trivial because the cost is implicitly evaluated along the optimal trajectory $\mathbf{q}^\star_{\boldsymbol{\alpha}}(t)$, which depends itself on the parameter $\boldsymbol{\alpha}$. More precisely, we have $C(\boldsymbol{\alpha}, \mathbf{q}^\star_{\boldsymbol{\alpha}}(t))$. This method is local but provides quite robust results for different points $\boldsymbol{\alpha}$. The results were in agreement with the first method described above. Table S2 reports the re-scaling vectors obtained from this analysis. By computing the gradient around the optimal cost combination found in the main text ($\boldsymbol{\alpha}^\star$ being the $\boldsymbol{\alpha}$ reported in Figure 5 of the main text), we could verify that the re-scaling vector we used agreed with the sensitivity analysis approach. Comparing the three first columns of Table S2 indicates that the two methods globally match well. The only exception concerns the coefficient corresponding to the torque component. This latter component was of magnitude 10^3 up to the sensitivity analysis rather than 10^4 as we used in the main text. When working at a totally different point, around $\boldsymbol{\alpha} = (1,\ldots,1)$, we found a very similar re-scaling vector, suggesting a certain robustness of the results. We note that, given the dependence of $\mathbf{r}$ on the point $\boldsymbol{\alpha}$ but also the initial parameters of the optimal control problems (e.g. starting posture, movement time etc.), only an order of magnitude can be reasonably obtained.

	Original values	At $\boldsymbol{\alpha}^\star$ ($\varepsilon = 10^{-2}$)	At $\boldsymbol{\alpha}^\star$ ($\varepsilon = 10^{-3}$)	At $\boldsymbol{\alpha} = \mathbf{1}$ ($\varepsilon = 10^{-2}$)
Hand jerk	1.00×10^{2}	9.58×10^{1}	8.62×10^{1}	1.59×10^{2}
Angle jerk	1.00×10^{1}	1.00×10^{1}	1.00×10^{1}	1.00×10^{1}
Angle acc.	1.00×10^{3}	9.05×10^{2}	9.90×10^{2}	9.49×10^{2}
Torque Chg.	1.00×10^{2}	1.59×10^{2}	1.04×10^{2}	4.30×10^{2}
Torque	1.00×10^{4}	1.28×10^{3}	1.41×10^{3}	2.92×10^{3}
Geodesic	1.00×10^{5}	1.17×10^{5}	1.17×10^{5}	2.31×10^{5}
Energy	1.00×10^{4}	9.68×10^{3}	1.13×10^{4}	1.64×10^{4}
Effort	1.00×10^{0}	1.78×10^{0}	1.63×10^{0}	3.26×10^{0}

Table S2: Re-scaling vector $\mathbf{r}$. For columns 2, 3 and 4, the average values across the 5 starting postures (P1 to P5) are reported. Values were found to be quite consistent across different starting postures (same order of magnitude). The only difference between columns 2 and 3 is the step size ε used to evaluate the gradient. The re-scaling vector has been normalized with respect to the angle jerk component. The vector $\boldsymbol{\alpha}^\star$ denotes the optimal weighting vector obtained from inverse optimal control (and, therefore, obtained when applying the inverse optimal control method with the re-scaling vector given in the 1st column).

References

[1] Guigon E, Baraduc P, Desmurget M (2007) Computational motor control: redundancy and invariance. J Neurophysiol 97:331–347. doi:10.1152/jn.00290.2006. URL http://dx.doi.org/10.1152/jn.00290.2006.

[2] Crevecoeur F, Thonnard JL, Lefèvre P (2009) Optimal integration of gravity in trajectory planning of vertical pointing movements. J Neurophysiol 102:786–796. doi:10.1152/jn.00113.2009. URL http://dx.doi.org/10.1152/jn.00113.2009.

[3] Gaveau J, Paizis C, Berret B, Pozzo T, Papaxanthis C (2011) Sensorimotor adaptation of point-to-point arm movements after space-flight: the role of the internal representation of gravity force in trajectory planning. J Neurophysiol doi:10.1152/jn.00081.2011. URL http://dx.doi.org/10.1152/jn.00081.2011.

[4] Gielen S (2009) Progress in Motor Control. A Multidisciplinary Perspective, Springer US, chapter Review of Models for the Generation of Multi-Joint Movements in 3-D. pp. 523–550.

[5] Calinon S, Guenter F, Billard A (2007) On learning, representing and generalizing a task in a humanoid robot. IEEE Transactions on Systems, Man and Cybernetics, Part B 37:286–298.

[6] Calinon S (2009) Robot Programming by Demonstration: A Probabilistic Approach. EPFL/CRC Press, 222 pp.
